# An impact of agronomic practices of sustainable rice-wheat crop intensification on food security, economic adaptability, and environmental mitigation across eastern Indo-Gangetic Plains

**DOI:** 10.1016/j.fcr.2021.108164

**Published:** 2021-06-15

**Authors:** J.S. Mishra, S.P. Poonia, Rakesh Kumar, Rachana Dubey, Virender Kumar, Surajit Mondal, S.K. Dwivedi, K.K. Rao, Rahul Kumar, Manisha Tamta, Mausam Verma, Kirti Saurabh, Santosh Kumar, B.P. Bhatt, R.K. Malik, Andrew McDonald, S. Bhaskar

**Affiliations:** a ICAR Research Complex for Eastern Region, Patna, Bihar, India; b Cereal Systems Initiative for South Asia (CSISA)-CIMMYT, Patna, India; c International Rice Research Institute, Los Banos, Philippines; d Birsa Agricultural University, Ranchi, Jharkhand, India; e Soil and Crop Sciences Section, School of Integrative Plant Sciences, Cornell University, Ithaca, NY, USA; f Indian Council of Agricultural Research, New Delhi, India

**Keywords:** Energy use, Global warming potential, Residue management, Resource conservation technologies, Rice-wheat-greengram system, Triple zero-tillage

## Abstract

In the eastern Indo-Gangetic Plains (EIGP), conventional rice-wheat system has led to a decline in productivity, input-use efficiency, and profitability. To address these, a four-year field study was conducted to evaluate the performance of tillage and crop establishment (TCE) methods in rice-wheat-greengram rotation. The treatments included: 1) random puddled transplanted rice (RPTR) - conventional-till broadcast wheat (BCW) - zero-till greengram (ZTG); 2) line PTR (LPTR) - conventional-till drill sown wheat (CTW) - ZTG; 3) machine transplanted rice in puddled soil (CTMTR) - zero tillage wheat (ZTW) - ZTG; 4) machine transplanted rice in zero-till wet soil (ZTMTR) - ZTW - ZTG; 5) system of rice intensification (SRI) - system of wheat intensification (SWI) - ZTG; 6) direct-seeded rice (DSR) - ZTW - ZTG; and 7) zero-till DSR - ZTW - ZTG. During the initial two years, conventional rice system (PTR) recorded a 16.2 % higher rice grain yield than DSR system. Whereas in the fourth year, the rice yields under DSR and PTR were comparable. As compared to SRI/SWI, the average wheat yield in ZT system was significantly high, whereas in rice, SRI/SWI system was comparable with CT system. ZTW after non-puddled rice was at par to CTW after PTR. The ZT wheat produced 4.6 % more yield than CT system. DSR production system consumed 6.8 % less water compared to transplanted system. On the system basis, 10.8 % higher net returns were recorded with CA-based system compared to conventional system. The system energy productivity under CA-based production system was 14–36 % higher than PTR-based systems. CA-based system also led to 8–10 % lower global warming potential (GWP) than conventional methods. The current study indicated that as compared to conventional system, a significant gain in productivity, profitability and energy-use efficiency, and reduction in the environmental mitigation are possible with emerging alternative TCE methods. Long-term expansion and further refinement of these technologies in local areas need to be explored for the second green revolution.

## Highlights

• Different crop establishment methods were compared with traditional conventional tillage (CT) in rice-wheat cropping system.
• Net returns and benefit cost ratio were increased by 11 and 28 % under conservation agriculture (CA) -based systems than CT.
• System productivity was at par while earthworm population was two times higher under CA-based production system than CT.
• CA-based systems had 15 % less energy input and 14–36 % higher energy productivity than CT.
• Direct seeded rice consumed 6.8 % less water and had 56.2 % less methane emission than puddled transplanted rice.

## Graphical abstract

**Figure:**
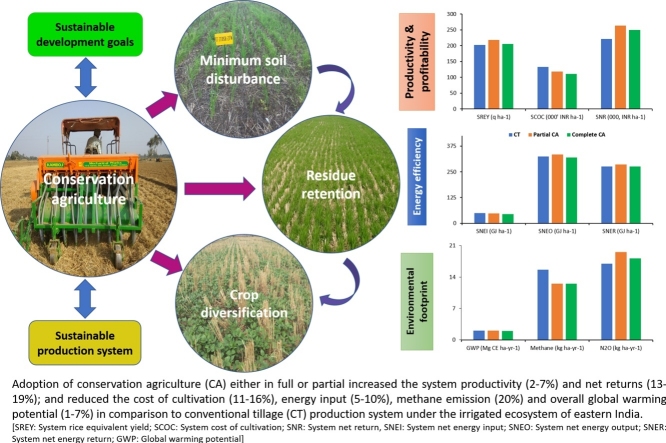

## 1 Introduction

Rice-wheat cropping system (RWCS) is the largest agricultural production system, extended over nearly 14 million ha in the Indo-Gangetic Plains (IGP) of South Asia (Alam et al., 2016). Although RWCS is vital for ensuring future food security and livelihood of millions of people in South Asia (Kumar et al., 2018), the path to success has been affected by declining soil health (Mondal et al., 2020), groundwater resources (Bhatt et al., 2016), monocropping of cereal-cereal system, increasing climate variabilities (Jain et al., 2014), and changing socio-economic dynamics (Dubey et al., 2020).

The EIGP has the majority (∼90 %) of marginal farmers, and the per capita income of INR 62,631 (US$ 835) per household per annum is much lower than the national (India) average of INR 94,130 (US$ 1256) (Mishra et al., 2020). Rice-wheat-summer fallow production system is extensively practiced by the farmers of this region. In this system, rice is conventionally grown by manual transplanting of 25–30 days old seedlings into puddled soil during wet season (June to October), and requires a large amount of water. The process of puddling requires ∼200−250 mm water (Rashid et al., 2018), and it is both labour and energy-intensive (Islam et al., 2020). Besides, the beneficial effects of soil puddling on rice through improved water retention, better weed control, quick seedling establishment and improved nutrient availability (Islam et al., 2020), it also deteriorates the physico-chemical (Mondal et al., 2019) and biological properties of the soil, and adversely affects crop growth and productivity of post-rice crops (Kumar et al., 2018). In the subsequent winter season (November-March) wheat is grown with repeated dry tillage by broadcasting wheat seeds and mixing the seed at the shallow depth by one or two passes of rotavator, resulting in poor crop establishment and lower yields (Paudel et al., 2020). After wheat harvest, fields remain fallow during the summer for a period of 75–80 days (April-June), and summer ploughing to destroy the insect pests and weeds, is a common practice by most of the farmers during fallow period.

In order to ensure the food security of the nation, eastern India has been targeted for ushering second Green Revolution. At present in RWCS, most of the rice area in EIGP is covered by long-duration (150–160 days) paddy varieties coupled with late transplanting. Late harvesting of rice, and longer turnaround period results in delayed wheat sowing with lower yield and quality due to terminal heat stress during grain filling (Jain et al., 2017). Tripathi et al. (2005) also reported an average yield loss of 27 kg ha^−1^ day^−1^ with delay in wheat planting beyond November 15. With increased use of combined machines for harvesting rice and wheat in the region, large amount of crop residue is left in the fields. According to one estimate, RWCS alone contributes ∼33 % of the total crop residue (∼ 620 million tons) generated every year in the Indian IGP (Raghuveer-Singh et al., 2019). Management of the loose and scattered rice residue after combined harvest is a major challenge as it obstructs the tillage operations and seeding of succeeding wheat crop (Sidhu et al., 2007). In order to clean the fields for sowing of wheat after rice, further greengram after wheat harvest, local farmers quickly burn the left-over rice/wheat residue, which is becoming a common inexpensive phenomenon in EIGP (Jain et al., 2014). It has been estimated that burning of one ton of rice residue accounts for a loss of 5.5 kg N, 2.3 kg P, 25 kg K and 1.2 kg S besides, organic carbon (NPMCR, 2014). Crop residue burning results in environmental pollution including greenhouse gas (GHG) emission, loss of earthworms/microbial population, lowering of soil nutrients and organic humus content in the top soil, which in long run reduces the soil fertility and sustainability (Singh et al., 2020b). Stopping crop residue burning will not only counteract the GHG emission (6266 Gg yr^−1^) but can also improve soil health when incorporated into the soil (Benbi, 2018).

Conservation Agriculture (CA)-based management paradigm (i.e., minimal soil movement, retaining crop residue cover on soil surface, and use of diversified crop rotation) adopted by the farmers in South Asia (Mishra et al., 2020) has prompted scientists to revisit and provide solutions to many emerging challenges (Ranaivoson et al., 2017). The CA practices and cropping systems followed elsewhere are diverse than those adopted in the EIGP. While puddling and transplanting are popular in rice, ZT with or without crop residue retention is practiced in succeeding winter crops indicating that the protocols of CA are partially adopted in the EIGP.

The beneficial effects of CA are largely attributed to the permanent crop residue cover on the soil surface (Ranaivoson et al., 2017). The positive effects of crop residue depend upon its quantity, quality, and placement. The adoption of CA not only provides extra inputs of nutrients but also prevents its loss from local ecosystem (Alves et al., 2010). New facts about gradual N-release from legume residue are even more compelling (Alves et al., 2010). The efficiency of a zero-tillage production system depends on the amount and distribution of plant residue left on soil surface (Samal et al., 2017). Zero tillage with residue retention offers a yield advantage of 5.8 %, improved water use efficiency by 12.6 %, an increase in net income of 25.9 %, and a reduction of 12.33 % in global warming potential (Jat et al., 2020). Despite several benefits, there is a slow adoption rate of ZT in the EIGP because of the limited access to required inputs (seeding machinery, herbicides, etc.), and old mindset of farmers (Keil et al., 2015; Lal, 2007).

The vulnerability of smallholder subsistence farmers (Thierfelder and Mupangwa, 2014) to global climate change (Wassmann et al., 2009) is expected more with rice yields projected to reduce about 3.2–22 % with an increase in air temperature (1−4 °C) by the end of this century as per simulated model (Zhao et al., 2017). It has been estimated that climate change may likely reduce the rice-wheat system productivity by about 4 % under farmers’ practice across eastern IGP (Tesfaye et al., 2019). As the average temperature in this region rises, early sowing of wheat using resource conservation technologies (RCTs) will become even more important (Dubey et al., 2020). It is not enough to increase the yield of RWCS, a third crop must be offered to the smallholders of EIGP for achieving a 300 % cropping intensity. The time saving through CA-based interventions will facilitate early wheat sowing and the timely sowing of greengram in succession. Inclusion of grain legumes into crop sequences helps in the accumulation of soil carbon and nitrogen (Borase et al., 2020), and reduces soil CO_2_ emission (de Araújo Santos et al., 2019d) under ZT production system. Diversification of rice-wheat cropping system with greengram would not only improve the soil health and farm income, but also reduce the water use and increase the adaptability to heat and water stress (Pathak, 2010).

New facts and crop establishment (CE) methods may shift the economic fundamentals in favour of sustainable intensification of RWCS. Some CE methods including zero-tillage with crop residue retention on the soil surface, mechanical rice transplanting, system of rice intensification (SRI), and DSR have their strengths and weaknesses (Jat et al., 2019b). Considering all these, triple zero-till cropping (ZT DSR-ZT wheat-ZT greengram) under the irrigated agro-ecosystem of EIGP may emerge as more sustainable RWCS for smallholder farmers.

Most of the studies in EIGP were conducted on zero-tillage based on a single crop in rice-wheat production system. Hence, the present study was focused to identify the suitable tillage and crop establishment practice to improve the productivity and profitability of rice-wheat-greengram system. In addition, some important CA-based parameters like energy use, and how efficient use of energy can help to reduce the environmental footprints. We hypothesized that CA-based tillage and crop establishment methods would improve crop yields, water productivity, net income, and energy efficiency; and reduce the environmental footprint.

## 2 Materials and methods

### 2.1 Experimental site and soil

**Fig. 1:**
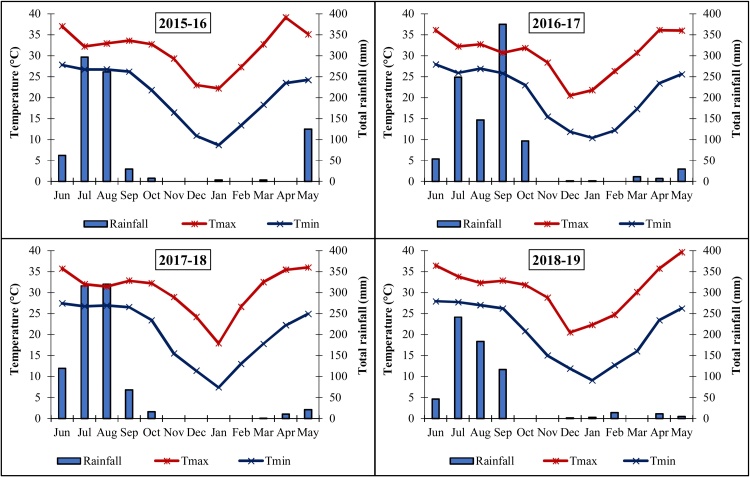
The mean monthly maximum temperature (Tmax °C), minimum temperature (Tmin °C) and total monthly rainfall (mm) for 4 crop years (2015–16 to 2018–19).

The field experiments were carried out at the research farm (25°35*'* N, 85°05*'* E, and 51 m above mean sea level) of ICAR Research Complex for Eastern Region, Patna, Bihar, India (Suppl. Fig. 1) during 2015–2019. The study continued for 48 months, where rice was grown during wet season (June-October), and wheat (November-March) and greengram (April-June) during dry seasons. The climate of the region is sub-tropical hot and humid, with an average annual rainfall of 1167 mm (70–75 % of which is received during July to September months), minimum temperature of 7.4–10.4 °C in January and maximum temperature of 35.1–39.6 °C in May. The annual mean humidity of the region was 67.2 %, being highest (80.5 %) and lowest (50.0 %) during September and April, respectively. Mean monthly temperature and monthly rainfall during the study period (2015−16 to 2018−19) are depicted in Fig. 1. The soil at the study site had a silty loam (Vertic Endoaqualfs) texture in the 0−15 cm surface layer with pH 7.22 and electrical conductivity (EC) of 0.17 dS m^−1^. The soil physical and chemical properties at the beginning of the experiment are presented in Suppl. Table 1.

### 2.2 Treatment details and experimental design

The experiment was initiated in June 2015 with rice crop after the harvest of wheat. The entire experimental area was laser-levelled in May 2015 before the start of the experiment. Seven combinations of tillage and crop establishment methods (TCE) for a rice-wheat-greengram cropping system were evaluated in a randomized complete block design with three replications. Each plot measured 8.1m × 20m. The seven TCE treatments were: 1) puddled random transplanted rice (RPTR) - conventional till (CT) broadcast wheat (BCW) - zero-till greengram (ZTG); 2) puddled line transplanted rice (LPTR) - CT drilled wheat (CTW) - ZTG; 3) puddled machine transplanted rice (CTMTR) - zero-till wheat (ZTW) - ZTG; 4) zero-till machine transplanted rice (ZTMTR) - ZTW - ZTG; 5) system of rice intensification (SRI) - system of wheat intensification (SWI) - ZTG; 6) CT direct-seeded rice (CTDSR) - ZTW - ZTG; and 7) zero-till DSR (ZTDSR) - ZTW - ZTG. Treatment notations and details of the management practices are presented in Table 1.

**Table 1:** Description of treatments/tillage and crop establishment practices adopted in the rice-wheat-greengram rotation from 2015-16 to 2018-19.

Parameter	Crop	RPTR-BCW-ZTG	LPTR-CTW-ZTG	CTMTR-ZTW-ZTG	ZTMTR-ZTW-ZTG	SRI-SWI-ZTG	CTDSR-ZTW-ZTG	ZTDSR-ZTW-ZTG
		T1	T2	T3	T4	T5	T6	T7
Tillage	Rice	Cultivator: 2 passes (dry tillage: DT)			Zero-till (flooding before transplanting)	Cultivator: 2 passes (DT)	Cultivator: 2 passes	Zero-till
		Rotavator: 1 pass (wet tillage: WT)				Rotavator: 1 pass (WT)		
	Wheat	Cultivator: 2 passes		Zero-till		Cultivator2 passes:	Zero-till	
		Rotavator: 1 pass				Rotavator: 1 pass		
	Greengram	Zero-till						
Crop establishment	Rice:							
	Transplanting/ Seeding	Manual	Manual	Machine	Machine	Manual	Drill seeding	Drill seeding
	Seedling age	25 days	25 days	18 days	18 days	12 days	–	–
	Spacing (cm)	Random	20 × 15	23 × 14	23 × 14	25 × 25	22.5 cm row spacing	22.5 cm row spacing
	Wheat	Broadcasting	Drill seeding	Drill seeding	Drill seeding	Manual	Drill seeding	Drill seeding
	Greengram	Drill seeding with Happy Seeder						
Residue management	Rice	∼30 % mixed		∼30 % retained		∼30 % mixed	∼30 % retained	
	Wheat	∼30 % retained						
	Greengram	100 % incorporated						

### 2.3 Crop management

#### 2.3.1 Rice

Irrespective of the treatments, a medium-duration (135 days) hybrid rice ‘Arize 6444 Gold’ purchased from local market, was used throughout the study period. Direct-seeded rice (DSR) was drilled in rows at 22.5 cm apart during 3rd week of June every year using Zero-till Happy Seeder having an inclined-plate seed metering system (Dasmesh, Malerkotla, Sangrur, Punjab, India) at a seed rate of 25 kg ha^−1^. The seeding depth of 3−4 cm was maintained for all DSR (ZT/CT) treatments using a depth control system of the seeder. Nurseries for PTR, MTR and SRI were sown on the same day of DSR seeding, with the recommended package of practice (Singh et al., 2020a). For MTR, a mat-type nursery was raised using a seed rate of 20 kg ha^−1^. A mixture (4:1) of fine sieved soil and farmyard manure (FYM) was prepared and spread on a polythene sheet with a thickness of 1.5–2.0 cm on raised beds (Singh et al., 2020a). The moisture was maintained initially by regular spraying of water, and later by light irrigations. The polythene sheet acted as a barrier between rice seedlings roots and underlying soil, and does not allow the roots of rice seedlings to penetrate the below soil. Hence, a dense mat of roots is formed on the sheet that can easily be uprooted for mechanical transplanting (Singh et al., 2020a). For PTR and SRI, non-mat nurseries were prepared with a seed rate of 15 and 7 kg ha^−1^, respectively. In SRI, 12-day-old seedlings were uprooted from nursery and transplanted manually with single seedling at 25 × 25 cm spacing within 30 min of uprooting. For PTR, 25-day-old seedlings were transplanted manually in puddle field at 20 × 15 cm spacing with 2−3 seedlings hill^−1^, whereas for MTR 18-day-old seedlings were transplanted with an 8-row self-riding type paddy transplanter (VST Pvt Ltd, Bengaluru, Karnataka, India) at 23 × 14 cm spacing (Singh et al., 2020a). The details of tillage and crop establishment operations adopted are given in Table 1.

The rice crop was fertilized with 120 kg N as urea and di-ammonium phosphate (DAP), 60 kg P_2_O_5_ as DAP, 60 kg K_2_O as muriate of potash (MOP) and 25 kg ZnSO_4_ per hectare (Jat et al., 2014). Full dose of phosphorus, potash, ZnSO_4_ and 1/3 rd of nitrogen (minus N applied as DAP) was applied at the time of sowing/transplanting. For transplanted rice (PTR/MTR/SRI), basal dose of fertilizers was applied at final puddling just before transplanting. For DSR, these were drilled at the time of sowing using Zero-till Happy Seeder. Remaining 2/3 rd of nitrogen was applied in two equal splits at mid tillering and panicle initiation stages of crop (Kumar et al., 2017).

To manage the existing weeds (grassy, broad-leaved and sedges) in ZT plots (ZTDSR/ ZTMTR), glyphosate at 1.0 kg a.i. ha^−1^ was applied one week before sowing/transplanting. In DSR, pendimethalin 1.0 kg a.i. ha^−1^ as pre-emergence at one day after seeding (DAS), and bispyribac-sodium at 25 g a.i. ha^−1^ at 20−25 DAS were applied to manage the weeds. In PTR and MTR, pretilachlor at 0.75 kg a.i. ha^−1^ was applied at 2−3 days after transplanting (DAT) followed by bispyribac-sodium at 25 g a.i. ha^−1^ at 20−25 DAT (Singh et al., 2020a). Herbicides were applied with 500 L ha^−1^ of water by using a knapsack sprayer fitted with flat-fan nozzle. Additionally, one hand weeding was also done to remove any escaped weeds. In SRI, weeds were managed by using a cono-weeder twice at 15–20 DAT and 30–35 DAT. No major incidence of insect-pest and diseases was observed in rice.

All DSR plots (ZT/CT) were sown after receipt of pre-monsoon rains and when there was sufficient soil moisture for germination. Subsequent irrigations were given when dry-spells exceeded more than one week and appearance of hairline soil cracks. In the event of normal rainfall, no irrigation was applied. For transplanted rice (PTR/MTR/SRI), puddling was done in flooded field with ∼7-cm water using a rotavator. Transplanting of rice seedlings was done with ∼5-cm standing water followed by continuous flooding with ∼ 5-cm for the first 8–10 days to facilitate proper crop establishment. Subsequent irrigations were applied at disappearance of flood water and appearance of hairline soil cracks as per the criterion of alternate wetting and drying (AWD) (Gathala et al., 2013). All the plots (DSR and transplanted) were irrigated at the same time depending on rainfall events. The number of post-sowing/transplanting irrigations varied from 3 to 5 in different years depending upon the amount and distribution of rainfall. Irrigation was applied through polyvinyl chloride pipes of 10-cm diameter. In each irrigation, ∼ 5-cm water was applied. The amount of irrigation water applied to each plot was measured using a water meter (Dasmesh Water Meters, Amritsar, Punjab, India). Rainfall data were recorded using a rain-gauge installed in the automatic weather station at the experimental site. The total quantity of water applied was calculated by adding up the amount of water applied through irrigation and rainfall (I + R). The quantity of irrigation water applied and water productivity were computed for each treatment. The source of irrigation water was always ground water. The amount of irrigation water applied was quantified, and water productivity (WP _I+R_) was computed as follows (Choudhary et al., 2018).

(1) Volume of irrigation water (kilolitre ha^−1^) = [(Final water meter reading-Initial water meter reading)/Plot area in m^2^] X 10,000

(2) Irrigation water (ha-mm) = Volume of irrigation water (kilolitre ha^−1^)/10

(3) Total water productivity (WP _I+R)_ (kg grain m^−3^ of water) = Grain yield (kg ha^-1^)/[Irrigation water applied (m^3^) + rainfall received by crop (m^3^)] ha^-1^

Where, 1 ha-mm irrigation depth = 10 kilolitre = 10 m^3^; 1 m^3^ = 1000 L

Economic water productivity, defined as the value derived per unit of water used, was estimated by multiplying the WP (I + R) values with minimum support price (MSP) of respective crops.

Yield attributes of rice, i.e., panicle length (cm), number of panicles m^−2^, number of grains panicle^-1^ and 1000-grains weight (g) (at 14 % moisture content) were recorded at maturity. The number of panicles were counted using 1 m^2^ quadrate from four places in each plot and averaged. Panicle length (cm), number of grains per panicle and 1000-grains weight (g) were determined from ten randomly selected panicles in each plot. Matured rice under DSR was harvested during last week of October (28–31 October), whereas the TPR was harvested around one week later (7–10 November) in each year. A net area of 8 m × 2 m (16 m^2^) from the centre of each plot was harvested for recording grain and straw yields. Irrespective of the treatments, crop was harvested manually with sickle at ∼30 cm height from ground level, and threshed manually after proper sun drying. Grain yield was adjusted at 14 % grain moisture content.

#### 2.3.2 Wheat

After harvest of rice, the most popular wheat cultivar ‘HD 2967’ procured from Borlaug Institute for South Asia (BISA), Pusa, Bihar, was grown throughout the study. Although the DSR was harvested about one week earlier to TPR, subsequent wheat was sown during 3rd week of November at the same time in all the treatments. Before sowing, CT and SWI (System of Wheat Intensification) plots were prepared using the conventional tillage practices (Singh et al., 2020a), whereas ZT plots were sown without tillage in anchored rice crop residue (Table 1). CT and ZT wheat plots were drilled in rows 22.5 cm apart at 100-kg seed ha^−1^ using Happy Seeder having inclined-plate seed metering system. CT-broadcast (BCW) was established by manual broadcasting and mixing with rotavator with 20 % higher seed rate (120 kg ha^-1^). The SWI wheat was established manually by dibbling method keeping seed rate of 25 kg ha^-1^. The seeding depth of ∼5 cm was maintained for all treatments.

The wheat crop was fertilized with 120 kg N as urea and DAP, 60 kg P_2_O_5_ as DAP, 60 kg K_2_O as MOP per hectare (Jat et al., 2019b). Full dose of phosphorus and potash and 1/3 of nitrogen (minus N applied as DAP) were applied at the time of sowing. For drill seeding (CT/ZT), these were applied at the time of sowing using zero-till Happy Seeder, whereas, for broadcast/ SWI, basal dose of fertilizers was applied at final field preparation just before sowing. Remaining 2/3 nitrogen was applied in two equal splits at crown root initiation (CRI) and maximum tillering stages of crop, which coincided with the first and third irrigations, respectively.

Wheat crop was established with pre-sowing irrigation. Irrigation was applied through polyvinyl chloride pipes of 10 cm diameter. In addition to pre-sowing irrigation, four irrigations were applied in all the plots coinciding with critical growth stages of wheat (CRI, tillering, flowering and grain filling stages). In each irrigation, ∼ 5 cm water was applied. The amount of irrigation water applied to each plot was measured using a water meter. In all ZT plots, glyphosate at 1.0 kg a.i. ha^−1^ was applied before sowing to kill the emerged weeds. Additionally, ready-mix solution of sulfosulfuron (75 %WG) + metsulfuron methyl (5 %WG) @ 32 (30 + 2) g a.i. ha^−1^ was applied as post-emergence (25 days after sowing) in all the plots to control annual grasses and broad-leaved weeds. Herbicides were applied with 500 L ha^−1^ of water by using a knapsack sprayer fitted with flat-fan nozzle.

Yield attributes of wheat, i.e., spike length, number of spikes m^−2^, number of grains spike^-1^ and 1000-grains weight (g) (at 12 % moisture content) were recorded at maturity. The number of spikes were counted using 1 m^2^ quadrate from four places in each plot and averaged. Spike length, number of grains per spike and 1000-grains weight (g) were determined from ten randomly selected spikes in each plot. Wheat was harvested during the first week of April each year. A net area of 8 m × 2 m (16 m^2^) from the centre of each plot was harvested for recording grain and straw yields. Irrespective of the treatments, the crop was harvested manually with sickle at ∼ 30 cm height from ground level, and threshed manually after proper sun drying. Grain yield was adjusted at 12 % grain moisture content.

#### 2.3.3 Greengram

Short duration (60–65 days) greengram cultivar ‘SML 668’ released from Punjab Agricultural University, Ludhiana, Punjab, India was grown initially during 2016 which was replaced by ‘Samrat’, released from ICAR-Indian Institute of Pulse Research, Kanpur in 2017. Seeds of both varieties were purchased from the local market. A pre-sowing irrigation was applied immediately after wheat harvest, and seeds were drilled without tillage in anchored wheat crop residue during second week of April. Sowing was done in rows at 22.5 cm apart at 30-kg seed ha^−1^ using Zero-till Happy Seeder having an inclined-plate seed metering system. The seeding depth of ∼5-cm was maintained for all treatments using depth control system of the seeder. In all plots, glyphosate at 1.0 kg a.i. ha^-1^ was applied before sowing to kill the emerged weeds. Pendimethalin at 1.0 kg a.i. ha^-1^ was applied as pre-emergence one day after seeding to control subsequent weed emergence. Crop received 18 kg N and 46 kg P_2_O_5_ ha^-1^ through DAP. Full dose of nitrogen and phosphorus was applied at the time of sowing through Happy Seeder. In addition to pre-sowing irrigation, two irrigations (at 25 and 45 DAS) were applied in all the plots. Irrigation was applied through polyvinyl chloride pipes of 10 cm diameter, and in each irrigation ∼5 cm water was applied. The amount of irrigation water applied to each plot was measured using a water meter. Two sprays of triazophos 40 EC @ 2.0 mL l^-1^ of water were done to check the infestation of whitefly (*Bemisia* spp.) to manage yellow mosaic disease infestation. A net area of 8 m × 2 m (16 m^2^) from the centre of each plot was used for grain yield estimation. Grain yield was reported at 12 % grain moisture content. After picking its matured pods twice (except in 2018, when only one picking could be done due to early monsoon rains in June), whole plants were retained, removed and ploughed down in ZTDSR, ZTMTR and CTDSR/PTR/SRI treatments, respectively. The retained greengram plants were desiccated with spray of paraquat (Gramoxone 24 % SL) herbicide at 0.48 kg a.i. ha^-1^ with 500 L ha^-1^ of water before ZTDSR seeding.

### 2.4 System productivity

To compare the productivity of rice-wheat-greengram cropping system, the grain yields of wheat and greengram were converted into rice equivalent yield (REY t ha^−1^) and calculated as sum of rice yield and REYs of wheat and greengram for each treatment (Mondal et al., 2020).

(4) REY of wheat = [(Wheat grain yield x MSP of wheat) / (MSP of rice)]

(5) REY of greengram = [(Greengram grain yield x MSP of greengram) / (MSP of rice)]

(6) System productivity = Rice grain yield + REY of Wheat + REY of Greengram

Where, MSP- Minimum support price of Govt. of India

### 2.5 Residue management and estimation of crop residue recycled

All previous crop residue (stubbles left after crop harvest) were either retained on the soil surface or incorporated with tillage as per treatment except for ZTMTR, where all the greengram residue were removed to facilitate machine transplanting. In conventional-till wheat plots (CTW, BCW and SWI), ∼ 30 % rice residue (20−25 cm long stubble) was incorporated, whereas, in zero-till wheat plots, it was retained on the soil surface in succeeding wheat crop. In greengram, ∼30 % wheat residue (lower part) was retained in all the treatments. In rice, 100 % greengram residue (after picking its matured pods in the field) was retained in ZTDSR and removed in ZTMTR (to facilitate the machine transplanting of succeeding rice seedlings), and incorporated in CTDSR/PTR, SRI treatments, respectively. Maximum residue contribution was from rice and greengram followed by wheat. A total of 29.23 to 46.86 Mg ha^−1^ (megagram per hectare) of crop residue were recycled under different TCE methods (Table 2). To estimate the quantity of crop residue recycled in each treatment, the leftover crop residue (∼ 30 cm height from ground level) of rice and wheat were cut from ground level using 1 m^2^ quadrate from four places in each plot and sun-dried to a constant weight and expressed on dry weight basis Mg ha^−1^. In case of greengram, total shoot dry weight excluding picked pods in 1 m^2^ quadrate area from four places in each plot was considered.

**Table 2:** Crop residue recycled (Mg ha^−1^) under different treatments over the years.

Treatments	2015−16				2016−17				2017−18				2018−19				Grand total (4 yr.)
	R	W	GG	Sys	R	W	GG	Sys	R	W	GG	Sys	R	W	GG	Sys	
RPTR-BCW-ZTG	4.7a	3.3a	4.6a	12.5a	3.3a	3.7a	4.1ab	11.2ab	4.1b	3.4a	3.8b	11.4a	3.4b	3.2ab	3.6ab	10.1a	45.2a
LPTR- CTW-ZTG	4.3a	3.5a	4.2a	12.0a	3.5a	3.4ab	4.5a	11.5a	4.3ab	3.5a	4.8a	12.5a	3.3b	3.3ab	3.5ab	10.1a	46.1a
CTMTR-ZTW-ZTG	4.6a	3.1a	4.2a	11.9a	3.4a	3.7a	4.2ab	11.3ab	4.1b	3.2a	4.1ab	11.4a	3.4b	3.4ab	4.1a	10.9a	45.4a
ZTMTR-ZTW-ZTG	4.3a	3.2a	0	7.5b	3.6a	3.9a	0	7.45c	3.9b	3.4a	0	7.3b	3.2b	3.8a	0	7.0c	29.2b
SRI-SWI-ZTG	4.2a	3.3a	4.3a	11.9a	3.5a	3.1b	3.8b	10.3b	4.8a	3.2a	4.3ab	12.3a	3.4b	2.5c	2.7c	8.6b	43.0a
CTDSR-ZTW-ZTG	4.9a	3.3a	4.3a	12.4a	3.2a	3.8a	4.0ab	10.9ab	4.8a	3.3a	4.4ab	12.5a	3.9a	3.5ab	3.6ab	11.0a	46.9a
ZTDSR-ZTW-ZTG	4.5a	3.4a	4.3a	12.2a	3.0a	3.7a	4.1ab	10.8ab	4.6a	3.5a	4.2ab	12.3a	3.3b	4.0a	3.2bc	10.5a	45.7a

R: Rice; W: Wheat; GG: Greengram; Sys: System; yr: year.

### 2.6 Soil temperature

Soil temperature was measured in wheat during the season of 2018−19 by using bent stem soil thermometers (Venus Professional Grade Meteorological Thermometers, Germany) graduated to 0.1 °C. The glass stem was bent 45° about 3 cm from the bulb. To check the uniformity of readings, the thermometers were kept in the same kind of environment before the experiment. In each experimental plot, one thermometer was installed in north-south direction in between the crop rows. A rod was pushed at 5 cm depth in the sidewall of undisturbed pit and the thermometer was installed gently into the hole. The pit was filled again carefully and manually added the crop residue on the soil surface. Soil temperature measurement started after germination of wheat and continued till harvesting of crop. Soil temperatures were manually recorded at 6:38 a.m. and 1:48 p.m. every day as per standard (Yadav et al., 2012). The daily soil temperatures were calculated by averaging the soil temperatures at 6:38 a.m. and 1:48 p.m. The daily temperature was grouped according to the different crop growth stages.

### 2.7 Measurement of photosynthesis

Photosynthesis (*P*n) was measured in rice (2017) and wheat during 2018−19 on fully expanded flag leaves at anthesis stage using a portable infrared gas analyzer (*LI-6400 Model, LICOR,* USA) (Dwivedi et al., 2018). Gas-exchange parameters were recorded between 10:00 and 11.30 h by providing an artificial light source of 1000 μmol (photon) m^−2^ s^−1^. *P*n was expressed as μmol (CO_2_) m^–2^ s^–1^.

### 2.8 Earthworm population

Earthworm populations were estimated in August 2018 by digging out one soil pit (0.3 m × 0.3 m × 0.3 m) in each plot and hand-sorting to collect and count the worms (Ashworth et al., 2017). Due care was taken to remove the soils attached with earthworms. The total counted earthworms were weighed immediately on an electronic balance. Earthworm biomass was expressed on a fresh weight basis.

### 2.9 Economic analysis

The economic analysis of rice-wheat-greengram cropping system was done during 2018−19 (after completion of 4th year of the experiment). Different economic indicators were calculated based on the existing market price of the inputs and outputs. The total cost of cultivation (TC) was calculated by taking in to account the variable cost (excluding land rent) including costs of seeds, fertilizer, pesticide, human labour, machines used for land preparation, nursery raising, transplanting, irrigation, fertilizer application, plant protection, weeding, harvesting, and threshing, etc.; and time required per hectare to complete an individual field activity in each treatment (Kumar et al., 2021). Fixed cost was not considered. The labour cost incurred on various field operations was based on the person-days ha^−1^ (8 h is equal to 1 person-day as per the labour law of the Indian government). The cost of labour was estimated by multiplying labour used in all operations with minimum wage rate as per Govt. of India’s labour law (Minimum Wage Act, 1948). Gross returns (GR) were computed by multiplying the grain yield (Mg ha^−1^) of each crop by the minimum support price (MSP) offered by the Government of India for rice (INR 17,500 Mg ha^−1^), wheat (INR 18,400 Mg ha^−1^) and greengram (INR 69,750 Mg ha^−1^), while straw value was calculated using current local market rates (Kumar et al., 2021). Net returns (NR) were calculated as the difference between GR and total cost (TC) (NR = GR-TC). Benefit: cost ratio was worked out by dividing gross returns with total cost (B:C ratio = GR/TC). The value of 1 US$ = 70.41 INR as per 2019 average exchange rate (https://www.exchangerates.org.uk/USD-INR-spot-exchange-rates-history-2019.html), was considered for economic analysis.

### 2.10 Energy estimation

To calculate the total energy (renewable and non-renewable) used in each crop, the energy equivalent (MJ unit^−1^) of each input was used (Suppl. Table 2). Renewable energy included human labour, farmyard manure, seeds, and water for irrigation; and non-renewable energy consisted of diesel, electricity, chemical fertilizers, pesticides, and farm machinery. Fuel consumed during each field operation, including land preparation, seeding, harvesting, and threshing was recorded to estimate energy consumption. Energy used in irrigation was computed by the number of hours, the electric pump was used. The farm produce (grain and straw yields) was also converted into energy in terms of energy output (MJ unit^−1^). Based on the energy equivalents of inputs and outputs, energy parameters were calculated by the following equations (Devasenapathy et al., 2009; Singh et al., 2020a).

(7) Energy use efficiency = Total energy output (GJ ha^−1^)/Total energy input (GJ ha^−1^)

(8) Energy productivity = Crop output (kg ha^−1^) / Energy input (GJ ha^−1^)

(9) Specific energy = Energy input (GJ ha^−1^) / Crop output (kg ha^−1^)

(10) Energy intensiveness = Energy input (GJ ha^−1^) / Cost of cultivation (INR ha^−1^)

(11) Net energy = Energy output (GJ ha^−1^) - Energy input (GJ ha^−1^)

### 2.11 Global warming potential (GWP)

Global warming potential (GWP) in terms of carbon dioxide equivalent was calculated based on emission factors (Suppl. Table 3) associated with agricultural inputs like diesel, electricity, fertilizers, herbicides, pesticides. Seasonal methane (for rice only) and nitrous oxide emission pattern for rice, wheat and greengram were estimated using emission factors. Methane is produced under anoxic condition with redox potential lower than −0.2 V. In our study under wheat and greengram the fields were never under anaerobic condition and redox potential was much higher making unfavourable condition for methane emission (Jat et al., 2019d). Moreover, no burning of crop residue was done in field during experiment period. Therefore, only CO_2_ and N_2_O are taken into consideration for greenhouse gas measurement of wheat and greengram. However, in case of rice CH_4_, N_2_O and CO_2_ emissions are taken into calculation. Methane emission factors were taken from the published literature and calculation based on our field inputs. The N_2_O estimation was calculated by emission factor (0.01) multiplied with quantity of N applied from all sources (fertilizers, residue) and converted into N_2_O emitted with multiplication with 44/28. The formula is given as follows (Tubiello et al., 2015).

(12) N_2_O emission (kg ha^−1^season^−1^) = Emission factor (0.01) × amount of external N applied (from residue, fertilizers etc. in kg/ha) × 44/28

Global warming potential was calculated as per Babu et al. (2020):

(13) GWP (kg CO_2_ eq. ha^−1^) = (emitted N_2_O × 298) + (emitted CH_4_ x 28) + emitted CO_2_

### 2.12 Statistical analysis

All the data on yield attributes, yield, water productivity, economics and energy were subjected to analysis of variance (ANOVA) for randomized complete block design using Statistix 8.1 statistical package (Analytical Software, Tallahassee, FL, USA; Luo et al., 2020). The differences between treatments means were compared using least significant difference (LSD) test at P < 0.05 (Gomez and Gomez, 1984).

## 3 Results and discussion

### 3.1 Weather

Mean meteorological parameters during cropping seasons were recorded at ICAR Research Complex for Eastern Region meteorological observatory nearby the experimental field in Patna, Bihar, India. Among the different weather parameters, rainfall pattern (both amount and distribution) was quite variable during the four years (2015−16 to 2018−19) of experimentation. The evidence for uncertainity of rainfall in the EIGP is a perennial problem and needs solution. The rainfall during the rice season (June–October) was the highest (921 mm) in 2016 followed by 840 mm in 2017 and 657 mm in 2015; whereas 2018 season received the lowest amount of rainfall (588 mm) (Fig. 1). Mean monthly maximum (31.8–37.0 °C) and minimum (20.8–27.9 °C) temperature during rice season were almost similar in all the four years of study (Fig. 1).

During wheat season (November to March) the rainfall received was almost negligible with maximum 17.6 mm during 2018−19. It shows that wheat cultivation in the region depends mainly on irrigation water. However, in wheat growing season, extreme temperature variations (sometimes high temperature at wheat sowing, and terminal heat stress at reproductive stage) were observed. Higher mean monthly maximum temperature was 2−3 °C higher during initial growth phase (in December) and during grain-filling stage (in March) 2015−16 and 2017−18 as compared to 2016−17 and 2018−19. The mean monthly minimum temperature (10.4 °C) in January 2017 was also 3 °C higher than that in January 2018 (7.4 °C) (Fig. 1). Likewise, in summer greengram cropping season the mean monthly maximum and minimum temperature were comparable in all the years ranging between 35−39 °C and 22−28 °C, respectively. The total amount of rainfall varied from 125 mm (2016) to 36 mm (2017). July to September months accounted for 73–87 % of the total yearly rainfall received during the study years (Fig. 1).

### 3.2 Yield attributes and yield

#### 3.2.1 Rice yield attributes

The pooled analysis of four years data showed that there was no significant (P < 0.05) effect of tillage and crop establishment methods on panicle length of rice (Table 3). However, number of panicles per square metre, number of grains per panicle and 1000-grains weight (g) were significantly altered by tillage and crop establishment methods. Zero-till mechanical transplanted rice (ZTMTR-ZTW-ZTG) being on par with direct-seeded rice (ZTDSR-ZTW-ZTG and CTDSR-ZTW-ZTG), recorded a significantly higher (9.7–25.6 %; P < 0.05) number of panicles per square metre (407) compared to transplanted rice (324–365) and SRI (371) (Table 3). System of rice intensification (SRI-SWI-ZTG), being at par with ZTDSR-ZTW-ZTG and RPTR-BCW-ZTG, recorded a significantly higher (17.7–36.9 %) number of grains per panicle (193) compared to other TCE methods (141–164). The RPTR-BCW-ZTG was recorded significantly higher (2.8–5.4 %) 1000-grain weight (23.38 g) than other TCE methods (22.18–22.75 g), while the lowest value with CTDSR-ZTW-ZTG (22.1 g).

**Table 3:** Yield attributes of rice/wheat as influenced by tillage and crop establishment methods in rice-wheat-greengram rotation (based on 4-year average, 2015-16 to 2018-19).

Treatments	Rice				Wheat			
	Panicle length (cm)	Number of panicles m^−2^	Number of grains panicle^−1^	1000-grain weight (g)	Spike length (cm)	Number of spikes m^−2^	Number of grains spike^−1^	1000-grain weight (g)
RPTR-BCW-ZTG	26.44a	324d	172ab	23.28a	11.10a	424b	49.30c	33.95b
LPTR- CTW-ZTG	26.19a	342cd	164bc	22.75b	11.16a	436b	55.34b	35.27ab
CTMTR-ZTW-ZTG	25.94a	365c	141c	22.57bc	11.02a	483a	55.76b	34.47b
ZTMTR-ZTW-ZTG	26.23a	407a	146c	22.29bc	11.56a	494a	61.51a	35.20a
SRI-SWI-ZTG	27.22a	371bc	193a	22.50bc	10.64a	444b	57.37ab	34.68b
CTDSR-ZTW-ZTG	26.81a	396ab	164bc	22.18c	11.36a	503a	53.06bc	35.58ab
ZTDSR-ZTW-ZTG	26.95a	399ab	177ab	22.42bc	11.00a	510a	57.24ab	36.75a

#### 3.2.2 Rice grain yield

The yield of rice was significantly (P < 0.05) affected by tillage and crop establishment methods. Grain yield of rice averaged over four years ranged from 6.27 Mg ha^−1^ under ZTDSR-ZTW-ZTG to 6.84 Mg ha^−1^ in LPTR- CTW-ZTG (Table 4). The grain yield during the first two years (2015 and 2016) was 16.2 % higher in transplanted system (PTR, MTR, and SRI) than direct-seeded rice (ZTDSR and CTDSR). During these two years, the lowest rice yield was recorded under ZTDSR-ZTW-ZTG production system. The initial reduction (14.15 %) in yield in zero-tillage practices compared to conventional practices (PTR, MTR) was due to the transition time in which soil takes time to stabilize attributing to increase soil stability, aggregate stability and increase soil carbon (Somasundaram et al., 2020). During the third year (2017), rice yield under SRI-SWI-ZTG was at par with CTDSR-ZTW-ZTG and ZTDSR-ZTW-ZTG production system (Table 4), while yield under transplanted rice (PTR and MTR) reduced by 21 %. In the last year (i.e., 2018), conventional puddled transplanted rice (LPTR and RPTR) yielded at par with direct-seeded rice (CTDSR and ZTDSR) but were significantly superior to other TCE methods. In the current study, LPTR produced a slightly higher (2.6–5.4 %) yield than RPTR across the years which is much less than 45 % reported by Awan et al. (2011). Line transplanting results in an optimum plant population which utilizes the resources more efficiently to produce a higher yield (Krishnan and Nayak, 2000).

**Table 4:** Grain yield of rice, wheat, greengram and system productivity under different establishment methods over the years.

Crop establishment methods	Grain yield of rice (Mg ha^−1^)					Grain yield of wheat (Mg ha^−1^)				
	2015	2016	2017	2018	Mean	2015−16	2016−17	2017−18	2018−19	Mean
RPTR-BCW-ZTG	7.15bcd	6.24a	5.70b	7.25ab	6.58ab	3.89b	5.09bc	3.94b	4.60abc	4.38b
LPTR- CTW-ZTG	7.34abc	6.48a	5.90b	7.64a	6.84a	4.45a	5.53ab	4.22ab	5.13ab	4.83a
CTMTR-ZTW-ZTG	7.51ab	5.44abc	5.69b	6.49b	6.28b	3.94b	5.45ab	3.99b	5.58a	4.77a
ZTMTR-ZTW-ZTG	7.68a	5.82ab	5.38b	6.19b	6.27b	4.23ab	5.83a	4.37a	4.24bc	4.67ab
SRI-SWI-ZTG	7.40ab	5.83ab	6.99a	6.19b	6.60ab	4.22ab	4.84c	4.43a	3.87c	4.34b
CTDSR-ZTW-ZTG	6.94cd	4.84bc	6.94a	7.29ab	6.50ab	4.16ab	5.61ab	4.22ab	4.76abc	4.69a
ZTDSR-ZTW-ZTG	6.84d	4.40c	6.70a	7.12ab	6.27b	4.93a	5.27abc	4.43a	4.95ab	4.90a

Crop establishment methods	Grain yield of greengram (Mg ha^−1^)					System productivity (REY Mg ha^−1^)				
	2016	2017	2018	2019	Mean	2015−16	2016−17	2017−18	2018−19	Mean
RPTR-BCW-ZTG	1.30a	0.87c	0.58c	1.96a	1.18b	14.87ab	14.96ab	12.20c	19.91b	16.18a
LPTR- CTW-ZTG	1.19a	0.99b	0.76b	2.30a	1.31ab	15.34a	16.11a	13.36b	22.21a	16.76a
CTMTR-ZTW-ZTG	1.31a	0.85c	0.72b	2.49a	1.34ab	14.19ab	14.49ab	12.75bc	22.29a	15.93a
ZTMTR-ZTW-ZTG	1.37a	0.83c	0.72b	2.13a	1.26ab	15.09a	15.21ab	12.86bc	19.13bc	15.57a
SRI-SWI-ZTG	1.36a	1.01b	0.70b	2.09a	1.29ab	15.04ab	14.77ab	14.47a	18.61c	15.72a
CTDSR-ZTW-ZTG	1.35a	1.21a	0.88a	2.24a	1.42a	13.96ab	15.34ab	14.83a	21.23a	16.34a
ZTDSR-ZTW-ZTG	1.26a	1.01b	0.84a	2.44a	1.39a	13.45b	13.78b	14.68a	22.06a	15.99a

REY = Rice equivalent yield.

The data pooled over four years indicated that the grain yield of rice was significantly (P < 0.05) higher by 9.09 % under LPTR-CTW-ZTG and by 5.65 % in SRI-SWI-ZTG compared to ZTDSR-ZTW-ZTG. It has been reported that yield loss ranges from 12 to 15% in DSR over PTR (Singh et al., 2020a; Xu et al., 2019). However, CTDSR (6.50 Mg ha^−1^) yielded at par to MTR (6.27–6.28 Mg ha^−1^), PTR (6.58–6.84 Mg ha^−1^) and SRI (6.60 Mg ha^−1^). From the present results, it can be derived that yield was lower in ZT rice plots compared to CT or PTR plots, and it was in agreement with previous study Chakraborty et al. (2017). This yield penalty in DSR can be attributed to soil sickness, higher weed growth because of favourable conditions due to alternate wetting and drying, moisture stress because of higher percolation rate (Chaki et al., 2021), this stress could be due to nematodes and rice mealybug, higher spikelet sterility, etc., which poses a challenge to maintain grain yield in rice (Kumar et al., 2018; Mishra et al., 2019; Riaz et al., 2018). These losses in DSR may be avoided by proper management of biotic and abiotic stresses. Paddy yield seems to converge towards LTPR with a yield gain of 9.1 % over other methods. In transplanted rice, puddling is beneficial for rice due to better weed control, reduction loss of water and nutrients through percolation, fast establishment of rice seedlings and better nutrient availability (Gathala et al., 2011a; Sharma et al., 2003).

#### 3.2.3 Wheat yield attributes

The ZTDSR-ZTW-ZTG, CTDSR-ZTW-ZTG, ZTMTR-ZTW-ZTG and CTMTR-ZTW-ZTG, did not have a significant effect within each other on the number of spikes (483−510 m^−2^) (Table 3), although SRI-SWI-ZTG, LPTR- CTW-ZTG and RPTR-BCW-ZTG reduced it (424 - 444 m^−2^). ZTDSR-ZTW-ZTG recorded the highest 1000-grain weight (36.75 g) and significantly higher number of spikes (494 - 510 m^−2^), whereas RPTR-BCW-ZTG produced the lowest grain weight (33.95 g). The current study showed that tillage practices affected yield-related parameters like number of spikes per square meter, number of grains per spike and 1000-grains weight (g) in wheat crop significantly. The current study was agreed with many previous results where zero tillage has increased grain yield by 11 % compared to conventional tillage (Zamir et al., 2010)

#### 3.2.4 Wheat grain yield

Wheat grain yield over four years ranged from 4.43 Mg ha^−1^ in SRI-SWI-ZTG to 4.90 Mg ha^−1^ under ZTDSR-ZTW-ZTG (Table 4). Wheat yield in SRI-SWI-ZTG treatment (4.22–4.84 Mg ha^−1^) was similar to ZTDSR-ZTW-ZTG (4.43–5.27 Mg ha^−1^) during first three years, but significantly lower (3.87 Mg ha^−1^) during 2018−19. The yield of ZT wheat grown after ZTMTR was 7–9.5 % higher than that obtained after CT MTR in all the four experimental years except in 2018−19. Wheat yields in SWI grown after SRI, and BCW after RPTR were significantly lower (8–11 %) than ZTW grown after DSR and CTW. Based on the four-year average, zero-till wheat (ZTW) produced 12.9 % more (560 kg ha^-1^) yield than SWI and 11.9 % higher than BCW production system, but it was at par with CTW (4.83 Mg ha^-1^) treatment. The current study clearly showed that as compared to other CE systems; zero tillage wheat is beneficial in terms of grain yield. Leaving crop residue on soil surface in ZT system protects soil from erosion, crusting and moisture loss through evaporation (Holland, 2004), and it improved carbon sequestration (Mondal et al., 2020), soil moisture conservation (Jat et al., 2020), enhanced rooting (Chaki et al., 2021), and reduces weed infestation (Mishra and Singh, 2012). Better wheat yield in ZTDSR - ZTW was observed due to saving of time in establishing crops by reducing tillage operations and better soil aeration and structure which facilitates good plant growth and yield (Singh et al., 2020b). In the present study, the wheat yields in CTDSR-ZTW-ZTG (4.69 Ma ha^−1^), LPTR-CTW-ZTG (4.83 Mg ha^−1^), CTMTR-ZTW-ZTG (4.77 Mg ha^−1^) production system was at par with ZTDSR-ZTW-ZTG (4.90 Mg ha^−1^), which also contrasted with previous studies (Kumar et al., 2018; Singh et al., 2020a) where yield under conventional wheat cultivation after puddled rice declined between 9–19 %. It calls for rigorous studies at farmers field with larger plot size so that the effect of smaller plots on sowing and irrigation operations can be minimized. The current study did not find any yield gain in SWI system (3.87–4.84 Mg ha^−1^) which was similar to the previous study Singh et al. (2020a). The conventional till drill-sown wheat (CTW) produced a 10.3 % higher yield than broadcast sowing (BCW) (Table 4). This yield loss can be attributed to poor seedling establishment, more prostrate plant growth and inefficient resource utilization in terms of water, sunlight, or nutrients in broadcasted seeds over drill-sown wheat (Collins and Fowler, 1992).

#### 3.2.5 Greengram grain yield

Greengram was drill-seeded in anchored wheat residue in all the TCE treatments. Grain yield of greengram over four years across the treatments ranged from 1.18 Mg ha^−1^ in RPTR-BCW-ZTG to 1.42 Mg ha^−1^ with CTDSR-ZTW-ZTG (Table 4). Grain yield was not affected by tillage and crop establishment in 2016 and 2019. However, during 2017 and 2018, it was significantly higher (39 and 52 %) in CTDSR-ZTW-ZTG compared to RPTR-BCW-ZTG. Grain yield was significantly (P < 0.05) higher (18–20 %) when grown under DSR-ZTW production system than RPTR-BCW system (Table 4). As greengram was zero-tilled uniformly in anchored wheat residue under all the treatments, differences in yield among the treatments are marginal. However, DSR (CT/ZT)-based CA system proved its superiority over conventional system (RPTR-BCW) due to conducive soil conditions under DSR that might have resulted in better crop growth and yield.

#### 3.2.6 Rice-wheat-greengram system productivity

Tillage and crop establishment methods had significant effects on system productivity in all the years of study (Table 4). The system productivity (rice-equivalent yield) over four years across the tillage and crop establishments methods ranged from 15.57 Mg ha^−1^ in ZTMTR-ZTW-ZTG to 16.76 Mg ha^−1^ under LPTR-CTW-ZTG production system. System productivity under Conservation Agriculture system (ZTDSR-ZTW-ZTG) was significantly lower by 12.3 and 14.5 % compared with conventional system (LPTR-CTW-ZTG) in the first two years (2015−16 and 2016−17), while it was significantly higher in the third year, and was at par in the fourth year. DSR (CT/ZT) based ZTW-ZTG production system, at par with each other, produced significantly higher REY than RPTR-BCW-ZTG system. Pooled average data of four-year, however, indicated that there was no significant effect of TCE methods on system productivity. In IGPs, rice is conventionally grown under puddled condition with intensive tillage and shifting to DSR from traditional puddling requires a shift in agronomic management practices, especially weeds and crop residue management (Gathala et al., 2011b). Therefore, zero-tillage yield declines in initial years in humid climatic condition and matches conventional tillage practices after 2−3 years. The same pattern was also seen during the experiment where system productivity was lower by 12–15 % in the initial two years. Later in third-year, system yield started to increase and was at par for the fourth year. Similarly, Jat et al. (2014) found that in rice-wheat rotation no-till with residue retention showed lower yield than conventional tillage and after 4–5 years, yield was equivalent in conservation as well as conventional tillage. The reason for a lower yield in initial years is ascribed to soil stabilization time in benefit return in terms of yield through enhanced soil aggregation, soil organic carbon, available soil moisture etc. (Pittelkow et al., 2015).

### 3.3 Soil temperature

**Fig. 2:**
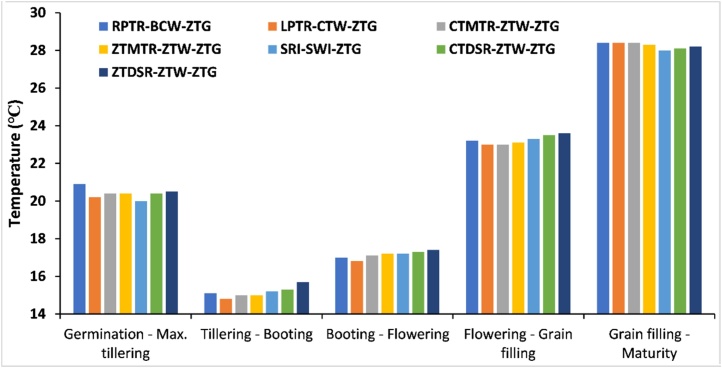
Mean soil temperature (0–5 cm depth) during the entire wheat season affected by different crop establishment methods.

A significant relationship was found between soil temperature and tillage systems. The mean soil temperature was higher in the BCW system (20.91 °C) than in zero-tillage production system from germination to grain filling stage (Fig. 2) in wheat. On average, there was 0.5 °C improvement in soil temperature under zero-till system with full residue when compared to LPTR-CTW-ZTG production system. Whereas later in reproductive stages, temperature was low as residue acted as mulch and provide thermal protection by restricting the movement of cold air between soil and atmosphere Therefore, zero tillage helped in impeding heat loss from soil in winter and hindered soil warming in summer compared to conventional tillage practices (Li and Sun, 2002)

### 3.4 Photosynthesis

**Fig. 3:**
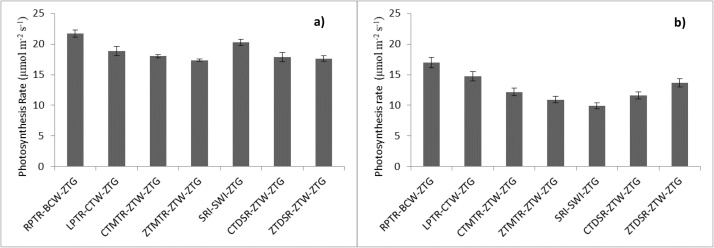
Changes in photosynthesis rate at anthesis stage under treatments in a) rice (2017) and b) wheat (2017–18).

Photosynthesis (Pn) in rice and wheat was estimated to assess the CO_2_ assimilation efficiency under different tillage and crop establishment practices. The current study results revealed that Pn was significantly influenced under different TCE practices. The rate of Pn in rice was the maximum (21.7 μmol m^−2^ s^−1^) under PTR followed by SRI (20.3 μmol m^−2^ s^−1^) (Fig. 3). The lowest Pn (17.4 μmol m^−2^ s^−1^) was recorded under zero-till machine transplanted rice (ZTMTR). The flag leaf photosynthesis in the case of wheat contributes by ∼ 30–50 % of the assimilates for grain filling (Sylvester-Bradley et al., 1990). Therefore, photosynthesis of flag leaves is the most important basis for the formation of the grain yield. Kumar et al. (2017) also reported that the rate of photosynthesis was higher in no-till (15.9 μmol m^−2^ s^−1^) as compared to conventional tillage (14.6 μmol m^−2^ s^−1^) in case of wheat, indicating the better moisture conserving ability and a lower rate of transpiratory water loss.

### 3.5 Earthworm population

**Fig. 4:**
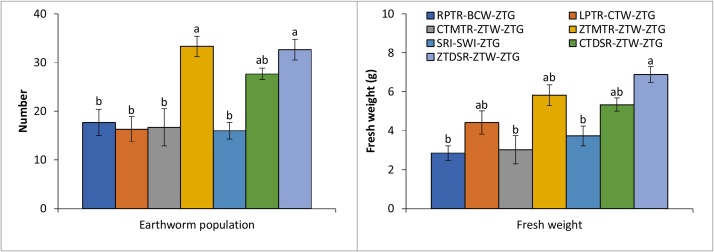
Earthworm population and fresh weight (30 × 30 × 30 cm soil depth) during the wet season (rice crop) as affected by different tillage and crop establishment methods.

Tillage and crop establishment methods had a significant (P < 0.05) effect on the population and fresh biomass of soil earthworm (Fig. 4). The earthworm population in CA-based production system (ZTDSR-ZTW-ZTG/ZTMTR-ZTW-ZTG) (32.67–33.33 numbers ft^−3^) was at par with partial-CA-based production system (CTDSR-CTW-ZTG) (27.67 ft^−3^) but it was significantly higher than the rest of the TCE methods (16.00-17.67 ft^−3^). Similarly, the fresh weight of earthworm under CA-based production system (ZTDSR-ZTW-ZTG) was significantly higher compared to conventional (RPTR-BCW-ZTG, CTMTR-ZTW-ZTG and SRI-SWI-ZTG) systems. Zero tillage has also shown powerful attraction for earthworm due to better soil aggregation; more photosynthetic carbon and available soil moisture in other contracting ecologies too (Ashworth et al., 2017).

### 3.6 Water use and water productivity

Of the total water used in rice-wheat-greengram cropping system, rice consumed 72 %, followed by wheat (16 %) and greengram (12 %) across the TCE methods. On a mean basis, rice grown under DSR production system consumed significantly less water by 6.8 % compared to transplant system (Table 5). The total water (I + R) uses in rice ranged from 10,808 m^3^ in CA-based system to 11,963 m^3^ in conventional and SRI-based systems. The water uses in wheat (2,410−2,658 m^3^) and summer greengram (1,749−1,994 m^3^) did not vary significantly with TCE methods. But as a whole system water productivity of rice-wheat-greengram was much higher compared to rice-wheat system alone (reported elsewhere) due to inclusion of greengram, which has a higher market value.

**Table 5:** Effect of tillage and crop establishment methods on water use and water productivity in rice-wheat-greengram system (2018–19).

Treatments	Total water (irrigation + rain) use (m^3^)				Water (irrigation + rain) productivity (kg grain m^−3^)				Economic water productivity (INR m^−3^)			
	R	W	GG	Sys	R	W	GG	Sys	R	W	GG	Sys
RPTR-BCW-ZTG	11503a	2658a	1994a	16154a	0.63ab	1.83bc	1.08a	1.26abc	11.0	33.7	75.3	22.1
LPTR- CTW-ZTG	11447a	2530a	1897a	15904abc	0.66a	1.93bc	1.17a	1.38ab	11.6	35.5	81.6	24.2
CTMTR-ZTW-ZTG	11708a	2325a	1749a	15776abc	0.56bc	2.41a	1.42a	1.41ab	9.8	44.3	99.0	24.7
ZTMTR-ZTW-ZTG	11471a	2547a	1910a	15928ab	0.54bc	1.68bc	1.11a	1.20bc	9.5	30.9	77.4	21.0
SRI-SWI-ZTG	11963a	2658a	1994a	16614a	0.52c	1.46c	1.05a	1.12c	9.1	26.9	73.2	19.6
CTDSR-ZTW-ZTG	10854b	2410a	1808a	15247bc	0.67a	1.98ab	1.24a	1.41ab	11.7	36.4	86.5	24.7
ZTDSR-ZTW-ZTG	10808b	2539a	1904a	15072c	0.66a	1.96ab	1.28a	1.45a	11.6	36.1	89.3	25.4

R: Rice; W: Wheat; GG: Greengram; Sys: System.

Total water use by ZTDSR-ZTW-ZTG production system (15,072 m^3^) was decreased significantly by 9.3 % as compared to SRI-SWI-ZTG. The total water consumed under DSR-based cropping system (15,072−15,247 m^3^) was at par, but significantly lower than the rest of the treatments (15,777−16,614 m^3^), which were at par with each other (Table 5). During the rainy season, where PTR is managed by puddling and flooding with standing water depth of ±5 cm, water use was around 7% higher than DSR where the field is only kept moist which are consistent with the results of (Kumar et al., 2018). In the literature, water saving of 20−25 % was noted with DSR over TPR (Kumar et al., 2018). Besides, ZT-DSR with residue retention in field additionally helped in soil moisture retention and further reduced the irrigation requirement.

The total water productivity (WP _I+R_) ranged from 0.52−0.67 kg grain m^−3^ in rice, 1.46–2.41 kg grain m^−3^ in wheat and 1.05–1.42 kg grain m^−3^ in greengram (Table 5). Water productivity is the ratio of per unit of water required to produce unit kg of yield. The WP of rice in DSR- based system was at par with puddled rice system, but increased significantly by 20 and 28 % compared to MTR and SRI based systems. Although yield under DSR was less than TPR due to less water use in DSR, the water productivity (0.66 kg grain m^−3^) was at par in both the systems. Secondly using zero tillage helped in additional water saving as residue retained not only acted as mulch but helped in reducing the evaporative loss. Improved WP in DSR (0.66−0.67 kg grain m^−3^) was due to reduced water usage while in ZTW (1.96–1.98 kg grain m^−3^) yield was higher. Since both the factor plays role in water productivity calculation hence, WP in DSR and ZTW was higher. In previous studies, zero tillage water productivity was 24 % more than conventional tillage practices (Das et al., 2014).

System water productivity (SWP) in terms of REY under the complete CA-based cropping system (1.45 kg grain m^−3^) was at par with partial CA-based system (1.41 kg grain m^−3^) and conventional system (1.26–1.38 kg grain m^−3^) but it was significantly (P < 0.05) higher compared to ZTMTR-ZTW-ZTG (1.20 kg grain m^−3^) and SRI-SWI-ZTG production system (1.12 kg grain m^−3^) (Table 5). The reason for higher system productivity in CA-based production system was due to comparative yields and less water use owing to the surface residue retention have been explained earlier in maize-based cropping system in NW India (Parihar et al., 2016).

### 3.7 Economic analysis

The cost of cultivation has differed significantly with respect to tillage and crop establishment methods. In rice, the total cost of production varied in the order of: SRI > LPTR > RPTR > CTMTR > ZTMTR > CTDSR > ZTDSR (Table 6). The total cost of production in CTDSR and CTMTR was lowered by INR 9,970 (US$ 142) and INR 5,460 ha^−1^(US$ 77), respectively compared to conventional system of PTR. Similarly, the total cost of production in ZTDSR was lower by INR 7,000 ha^−1^ (US$ 99) compared to CTDSR; and in ZTMTR by INR 7,200 ha^−1^ (US$ 102) compared to CTMTR. However, the total cost of production in SRI was at par with conventional system (PTR). The cost of production in zero-till wheat (ZTW) was lower by INR 7540 (US$ 107), INR 6,540 (US$ 93), and INR 4,500 (US$ 64) ha^−1^ compared to SWI, BCW and CTW. Since summer greengram was grown under zero-till system in all the treatments, cost of production (INR 32,400 ha^−1^) (US$ 460 ha^−1^) was the same. At system level, the highest cost of production was incurred in SRI-SWI-ZTG (INR 1,34,970 or US$ 1,917 ha^−1^) followed by RPTR-BCW-ZTG (INR 1,31,470 or US$ 1,867 ha^−1^) and LPTR-BCW-ZTG (INR 1,30,990 ha^−1^). The CA-based production system (ZTDSR-ZTW-ZTG/ZTMTR-ZTW-ZTG) was recorded the lowest cost of production (INR 1,08,730/1,13,040 ha^−1^) (US$ 1,544/1,605 ha^−1^) followed by CTDSR-ZTW-ZTG production system (INR 1,15,730 or US$ 1,644 ha^−1^). The cost reduction was principally due to a decrease in 4–5 tillage operation as reported elsewhere (Jat et al., 2019a).

**Table 6:** Effect of tillage and crop establishment methods on cost of production, gross returns, net returns and benefit: cost ratio in rice-wheat-greengram system (2018-19).

Treatments	Cost of production (INR ha^−1^*10^3^)				Gross returns (INR ha^−1^*10^3^)			
	Rice	Wheat	Green gram	System	Rice	Wheat	Greengram	System
RPTR-BCW-ZTG	58.44a	40.63ab	32.40a	131.47a	126.89ab	84.64c	136.71d	348.23c
LPTR- CTW-ZTG	60.00a	38.59b	32.40a	130.99a	133.70a	94.39b	160.43ab	388.52a
CTMTR-ZTW-ZTG	53.76b	34.09c	32.40a	120.24b	113.58c	104.51a	173.69a	391.76a
ZTMTR-ZTW-ZTG	46.56cd	34.09c	32.40a	113.04bc	108.33c	78.02cd	148.57bc	334.91d
SRI-SWI-ZTG	60.95a	41.63a	32.40a	134.97a	108.33c	71.21d	145.78cd	325.31d
CTDSR-ZTW-ZTG	49.25bc	34.09c	32.40a	115.73bc	127.56ab	87.58bc	156.24bc	371.40b
ZTDSR-ZTW-ZTG	42.25d	34.09c	32.40a	108.73c	124.60b	91.08b	170.19a	385.87a

Treatments	Net returns (INR ha^−1^*10^3^)				Benefit: cost ratio			
	Rice	Wheat	Greengram	System	Rice	Wheat	Greengram	System
RPTR-BCW-ZTG	68.43c	44.02c	104.31d	216.76c	2.17b	2.08bc	4.22c	2.82bc
LPTR- CTW-ZTG	73.70bc	55.81b	128.03b	257.53ab	2.23b	2.45b	4.95ab	3.21ab
CTMTR-ZTW-ZTG	59.82d	70.43a	141.28a	271.52a	2.11b	3.07a	5.36a	3.51a
ZTMTR-ZTW-ZTG	61.77cd	43.93c	116.17c	221.87c	2.33ab	2.29b	4.59bc	3.07b
SRI-SWI-ZTG	47.38e	39.58c	113.38c	190.34d	1.71b	1.75c	4.50bc	2.66c
CTDSR-ZTW-ZTG	78.33ab	53.50b	123.84bc	255.67b	2.59ab	2.57ab	4.82b	3.33ab
ZTDSR-ZTW-ZTG	82.36a	57.00b	137.79a	277.14a	2.95a	2.67a	5.25a	3.62a

INR: Indian Rupees.

Tillage and crop establishment methods had a significant (P < 0.05) effect on net returns from rice, wheat, greengram and at system level (Table 6). Net returns from rice was maximum (INR 82,360 or US$ 1,170 ha^−1^) under CA-based system (ZTDSR-ZTW-ZTG) followed by partial CA system (INR 78,330 or US$ 1,112 ha^−1^). The lowest NR (INR 47,380 or US$ 673 ha^−1^) in rice was realized under SRI followed by MTR (INR 59,820-61,770 ha^−1^) (US$ 850-877 ha^−1^). Zero-till wheat grown in CTMTR-ZTW-ZTG system fetched the maximum NR (INR 70,430 or US$ 1,000 ha^−1^) followed by CA-system (INR 57,000 or US$ 810 ha^−1^). The lowest NR in wheat (INR 39,580 or US$ 562 ha^−1^) was recorded under SWI. Broadcast wheat (BCW) fetched significantly lower NR by INR 11,790 ha^−1^ (US$ 167 ha^−1^) as compared to drill-sown wheat (CTW). Previous studies also reported higher net returns from zero tillage (Erenstein, 2002). During summer season, greengram under CTMTR-ZTW-ZTG production system being on a par with CA-based system gave higher net returns (INR 1,41,280 or US$ 2,007 ha^−1^) than the rest of the TCE methods (INR 1,04,310–1,28,030 ha^−1^) (US$ 1,481–1,818 ha^−1^). At the system level, the net returns ranged from INR 2,77,140 or US$ 3,936 ha^−1^ in CA-based production system to INR 1,90,340 or US$ 2,703 ha^−1^ under SRI-SWI-ZTG system. LPTR-CTW-ZTG method fetched the higher net returns of INR 40,770 ha^−1^ compared to RPTR-BCW-ZTG; and CTMTR-ZTW-ZTG by INR 49,650 or US$ 705 ha^−1^ compared to ZTMTR-ZTW-ZTG production system.

At the system level, the current study may draw comfort from the fact that productivity of CA based system may not improve every year, but net profits stayed higher than conventional methods. Long term prospects are also higher for improving water productivity and energy use. That is where the cost has come down. From this four-year study, it can be concluded that an amount of INR 19,610–60,400 ha^−1^ yr^−1^ could be saved by adopting CA-based system over conventional rice-wheat-greengram system. Similar results were obtained in a long-term (10 years) study on reducing tillage practices in rice-maize system that gave a profit of 225–1,028 US$ ha^−1^ yr^−1^ over conventional system (Jat et al., 2019b).

Among all the three crops, greengram had a higher B:C ratio (4.22–5.36) compared to rice (1.71–2.95) and wheat (1.75–3.07). Even after adjusting yield levels, all crop establishment methods of rice had B:C ratio in a range of 2.3–3.0 compared to B:C ratio of 1.7 from SRI). Zero-till wheat grown after CTMTR had the highest B:C ratio (3.07) and BCW has the lowest ratio of 2.1.

The lowest B:C ratio (1.75) in wheat was observed under SWI. At the system level, B:C ratio was in the order of ZTDSR-ZTW-ZTG (3.62) = CTMTR-ZTW-ZTG (3.51) = CTDSR-ZTW-ZTG (3.33) > ZTMTR-ZTW-ZTG (3.07) > SRI-SWI-ZTG production system (2.66). Higher B: C ratio in greengram was due to higher minimum support price (INR 69,750 t^−1^), which fetched higher gross returns than rice and wheat. Most important point to focus on is due to the intensification of the system net return of rice-wheat-greengram system as a whole has increased almost double to US$ 4,767 ha^−1^yr^−1^ compared to only rice-wheat system under Conservation Agriculture (US$ 2,300 ha^−1^ yr^−1^) as reported by Jat et al. (2014) in his studies based on 2013-dollar values or 2,900 ha^−1^yr^−1^ (Gathala et al., 2015).

### 3.8 Energy budgeting

This study compared the energy analysis between different crop establishment methods. The energy required in raising crops varied from 42.5 to 49.91 GJ ha^−1^. Among the crops under study, the energy required by rice was higher followed by wheat, while greengram used the lowest energy (Table 7). The highest energy consumption by rice mainly attributed to high fertilizer use, tillage practices and labour (Babu et al., 2020). The DSR-based cropping system had a significantly (P < 0.05) lower energy requirement compared to PTR-based system. The total energy requirement under Conservation Agriculture-based system was 14.87 % lower than that of conventional system.

**Table 7:** Effect of tillage and crop establishment methods on energy parameters in rice-wheat-greengram system (2018-19).

Treatments	Total energy input(GJ ha^−1^)				Total energy output(GJ ha^−1^)				Energy use efficiency				Energy productivity (kg GJ^−1^)			
	R	W	GG	Sys	R	W	GG	Sys	R	W	GG	Sys	R	W	GG	Sys
RPTR-BCW-ZTG	22.3a	19.9a	7.72a	49.9a	180a	139ab	33.8a	353a	8.09b	6.99ab	4.38ab	7.08b	0.34bc	0.26b	0.30b	0.40c
LPTR- CTW-ZTG	22.3a	19.9a	7.72a	49.9a	162ab	134ab	28.8c	325ab	7.28b	6.73bc	3.73c	6.51bcd	0.33c	0.22c	0.25c	0.45b
CTMTR-ZTW-ZTG	23.5a	17.9b	7.72a	49.1a	151b	144a	36.6a	332ab	6.44c	8.04a	4.74a	6.76bc	0.28d	0.31a	0.32a	0.45b
ZTMTR-ZTW-ZTG	20.8ab	17.9b	7.72a	46.5bc	143c	125bc	31.3b	299b	6.86bc	6.96ab	4.06bc	6.43cd	0.30d	0.24c	0.28bc	0.41bc
SRI-SWI-ZTG	21.6ab	19.4a	7.72a	48.7ab	149bc	117c	30.7bc	296b	6.88bc	6.04c	3.98bc	6.09d	0.29d	0.20c	0.27bc	0.38c
CTDSR-ZTW-ZTG	19.5b	17.9b	7.72a	45.1c	173ab	129abc	32.9b	336a	8.90a	7.23ab	4.27ab	7.44ab	0.37b	0.27b	0.29b	0.47ab
ZTDSR-ZTW-ZTG	16.89c	17.9b	7.72a	42.5d	164ab	140.6a	35.9a	341a	9.75a	7.85a	4.65a	8.02a	0.42a	0.28b	0.32a	0.52a

Treatments	Specific energy (GJ kg^−1^)				Energy intensiveness (GJ INR^−1^)				Net energy (GJ ha^−1^)			
	R	W	GG	Sys	R	W	GG	Sys	R	W	GG	Sys
RPTR-BCW-ZTG	1.70bc	1.95bc	3.35bc	1.95bc	0.38ab	0.49a	0.24a	0.38a	158.03a	119.05a	26.09ab	303.17a
LPTR- CTW-ZTG	1.90b	2.00ab	3.94a	2.11ab	0.37ab	0.51a	0.24a	0.38a	140.13bc	113.81ab	21.10c	275.04b
CTMTR-ZTW-ZTG	2.14a	1.70c	3.10c	2.05ab	0.44a	0.53a	0.24a	0.41a	127.58cd	125.99a	28.89a	282.46ab
ZTMTR-ZTW-ZTG	2.01ab	1.94bc	3.62ab	2.14ab	0.45a	0.53a	0.24a	0.41a	122.04d	106.80ab	23.60bc	252.438c
SRI-SWI-ZTG	2.00ab	2.23a	3.69ab	2.26a	0.35b	0.48a	0.24a	0.36a	127.24cd	97.53b	23.01bc	247.78c
CTDSR-ZTW-ZTG	1.55cd	1.88bc	3.44b	1.85bc	0.40ab	0.53a	0.24a	0.39a	153.93a	111.56ab	25.21ab	290.71ab
ZTDSR-ZTW-ZTG	1.42d	1.73c	3.16c	1.72c	0.40ab	0.53a	0.24a	0.39a	147.55ab	122.73a	28.15a	298.43a

R: Rice; W: Wheat; GG: Greengram; Sys: System; 1 US$ = 70.41 INR as per 2019 average exchange rate.

In the case of puddled transplanted rice and conventional wheat, the total energy input was higher; in which PTR took maximum labour (transplanting, weeding, harvesting, threshing) as well as a high amount of diesel used mainly for puddling which adds to the input energy (Mitra et al., 2018). Total energy requirement in rice and wheat (16.87 and 17.91 GJ ha^−1^) under CA based system (ZTDSR-ZTW-ZTG) was significantly lower by 24.28 and 9.86 % than conventional (PTR-CTW) system (Table 7). The total input energy in greengram (7.22 GJ ha^−1^) was similar in all the treatments. Reduced energy requirement in rice and wheat under zero-tillage was mainly due to less input in terms of labour, diesel used for tillage and water irrigation (Babu et al., 2020; Mitra et al., 2018). The energy output in rice, wheat and greengram under different treatments ranged from 142.87–180.31, 116.89–143.90 and 28.81–36.60 GJ ha^-1^, respectively. The energy output in rice (180.31 G J ha^-1^) under RPTR-BCW-ZTG treatment was at par to CTDSR-ZTW-ZTG (173.41 GJ ha^-1^) ZTDSR-ZTW-ZTG (164.41 GJ ha^-1^) and LPTR-CTW-ZTG (162.45 GJ ha^-1^), but significantly higher compared to other treatments. In wheat, CTMTR-ZTW-ZTG production system being at par with DSR-based and PTR-based systems recorded significantly higher (23 %) output energy over SRI-SWI-ZTG system. Biomass and straw yield in rice and wheat establishment methods were at par which is being reflected in terms of output energy.

In greengram, CTMTR-ZTW-ZTG (36.60 GJ ha^−1^) being at par with ZTDSR-ZTW-ZTG (35.87 GJ ha^−1^) and RPTR-BCW-ZTG (33.81 GJ ha^−1^) recorded significantly higher energy output compared to other treatments. The system output energy ranged from 296.48 GJ ha^−1^ under SRI-SWI-ZTG to 353.01 MJ ha^−1^ under RPTR-BCW-ZTG (Table 7). The higher energy output in RPTR-based system was due to higher rice straw yield. The energy output under CA-based production system (ZTDSR-ZTW-ZTG) was 15 % higher than SRI-SWI-ZTG system.

To judge the system efficiency economic analysis is determined. More efficient system is that which consumes less energy and gives a higher output (Yadav et al., 2017). To estimate which system is more energy-efficient different indices have been used. In general, the energy use efficiency (EUE) was found higher than those of only rice-wheat system followed elsewhere. The study results showed that the inclusion of legumes especially summer greengram is more efficient in terms of EUE (Soni et al., 2018). The EUE ranged from 6.44–9.75, 6.04–7.85 and 3.73–4.65 in rice, wheat and greengram, respectively (Table 7). The EUE of all three crops was significantly higher under CA-based cropping system (4.27–9.75) compared to conventional (LPTR- CTW-ZTG) (3.73–7.28) and (SRI-SWI-ZTG) (3.98–6.88) systems. The lowest EUE in rice (6.44) was recorded with CTMTR-ZTW-ZTG followed by SRI-SWI-ZTG (6.88) and ZTMTR-ZTW-ZTG (6.86) systems. Wheat in SRI-SWI-ZTG system (6.04), and greengram in LPTR- CTW-ZTG (3.73) had the lowest EUE. Energy use efficiency of the system varied from 6.09 to 8.02 being the lowest in SRI-based production system and the highest in CA-based production system. Similar results showed higher energy efficiency in zero-tillage followed by reduced and conventional tillage in pigeonpea-castor system (Pratibha et al., 2015). As energy use efficiency is the ratio of output energy to input energy, it can be derived that CA-based production system had lesser input energy and produced at par output energy compared to conventional system which enhanced its overall energy use efficiency (Jat et al., 2019c)

The current study results showed that energy productivity (EP) (kg grain produced per GJ energy consumed) in rice, wheat and greengram varied from 0.277−0.422, 0.200−0.312 and 0.254−0.325 kg GJ^−1^, respectively (Table 7). Here in the study, a significantly higher energy productivity in rice (0.422 kg GJ^−1^) was observed in the CA-based rice-wheat-greengram cropping system, which was 12.8 % higher than partial CA-based production system (CTDSR-ZTW-ZTG) and 29.8 % than conventional system (LPTR- CTW-ZTG). In wheat, CTMTR-ZTW-ZTG was recorded significantly higher EP (0.312 kg GJ^−1^) by 56 % over SRI-SWI-ZTG (0.20 kg GJ^−1^). The highest system EP in terms of REY (0.519 kg GJ^−1^) was achieved under CA-based production system, which was 10 % higher over CTDSR-based system and 14–36 % higher than PTR-based systems which also agreed with Jat et al. (2019c). Specific energy (calorific values required to produce 1 kg grain) ranged from1.42–2.14, 1.70–2.23 and 3.10–3.94 GJ kg^-1^ for rice, wheat and greengram, respectively (Table 7). Rice produced with CA-based system required 33.6, 29.0 and 25.3 % less specific energy compared to conventional CTMTR-ZTW-ZTG, SRI-SWI-ZTG and LPTR-CTW-ZTG systems, respectively. In wheat, CTMTR-ZTW-ZTG (1.70 GJ kg^−1^), being at par with ZTDSR-ZTW-ZTG (1.73 GJ kg^−1^) consumed significantly lower specific energy as compared to SRI-SWI-ZTG (2.23 GJ kg^−1^). A similar trend (3.10-3.94 GJ kg^−1^) was observed in case of greengram. The system specific energy was also recorded the lowest in CA-based production system (1.72 GJ kg^−1^), and the highest (2.26 GJ kg^−1^) under SRI-SWI-ZTG. These results (Table 7) indicated that various tillage and crop establishment methods had little difference in energy intensiveness, except in rice, where SRI-SWI-ZTG system recorded significantly lower value of energy intensiveness (0.35 MJ INR^−1^) compared to ZTMTR-ZTW-ZTG production system (0.45 GJ INR^-1^). The net energy (NE) values ranged from 122.04–158.03, 97.53–125.99 and 21.10–28.89 GJ ha^−1^ in rice, wheat and greengram, respectively (Table 7). In rice, RPTR-BCW-ZTG system (158.03 GJ ha^−1^) was at par with CTDSR-ZTW-ZTG (153.93 GJ ha^−1^) and ZTDSR-ZTW-ZTG (147.55 GJ ha^−1^) systems, but recorded significantly higher (29.5 %) NE over ZTMTR-ZTW-ZTG (122.04 GJ ha^−1^). The lowest NE in wheat (97.53 GJ ha^−1^) was attained with SRI-SWI-ZTG, which was 20.5 and 22.6 % lower than ZTDSR-ZTW-ZTG and CTMTR-ZTW-ZTG systems. In greengram, the highest NE (28.89 GJ ha^−1^) was obtained with CTMTR-ZTW-ZTG, and lowest (21.10 GJ ha^−1^) with LPTR- CTW-ZTG system. The system net energy ranged from 247.78 GJ ha^-1^ in SRI-SWI-ZTG to 303.17 GJ ha^-1^ in RPTR-BCW-ZTG. CA-based production system (298.43 GJ ha^−1^) produced 8.5 and 20.4 % higher net energy than LPTR- CTW-ZTG and SRI-SWI-ZTG systems. From all these energy observation, it is evident that CA-based production system with low input use and higher or at par output energy makes it more energy efficient system. Moreover, energy efficient CA-based system provides possibilities to intensify the rice-wheat system with the inclusion of legume crop as greengram which makes system more productive and efficient in terms of energy (Becerril and Rios, 2016).

### 3.9 Global warming potential

The global warming potential of different rice treatments showed that ZTDSR had the lowest and CTMTR, SRI, CTDSR, PTR had 15.5 11.3 %, 11.1 % and 9 % higher GWP (Table 8). With a change in rice tillage practices from PTR, ZTDSR has 56.2 % less methane emission. Under puddled soil methane is emitted under anaerobic condition in submerged soil by the methanogenesis process (Le Mer and Roger, 2001). As compared ZTDSR field were only kept moist which released less methane from soil (Jain et al., 2014). In a study, Gupta et al. (2016) reported that PTR treatments had 82–87 % higher emission compared to DSR. However, in the current study, the reduction was less (56.2 %) since DSR was under zero-tillage, where residue act as methane sources under irrigated condition. In case of nitrous oxide, emission was 28 % higher in ZTDSR compared to PTR which was because of alternate wetting and drying favouring nitrification and denitrification process (Jain et al., 2014). Major contributors towards higher GWP from PTR was due to diesel and methane emission, while fertilizer and herbicide emission rate was constant for both the systems. The present study results emphasized that puddling for which intensive tillage is required uses more diesel and provides anoxic environment for methanogens to act for higher GWP. Moreover, the GWP of zero-till wheat was 11.3 % lower than conventional till wheat. In wheat, major contributors to greenhouse gas emission are the use of electricity, diesel, fertilizer and herbicide use. Diesel was the most important factor for higher GWP of CTW, as in zero-till system, diesel use emitted 32.16 kg CO_2_ eq. emission compared to 150.08 kg CO_2_ eq. emission from CTW system (Table 8). Although N_2_O emission was 12 % higher in ZTDSR-ZTW-ZTG system compared to LPTR-CTW-ZTG as zero-till wheat. Results are similar to Bhatia et al. (2010) and Gupta et al. (2016) where ZTW had 8–12 % higher N_2_O emission compared to CTW. Higher N_2_O emission from ZTW may be due to higher compaction with higher bulk density and soil moisture which reduces oxygen diffusion rate favouring anaerobic condition under which more nitrous oxide is emitted (Gupta et al., 2016). Greengram under all the crop establishment methods was done under zero tillage condition with global warming potential of 241.81 (Table 8). The GWP of rice-wheat-greengram system averaged over four years varied from 1,925−2,114 kg CO_2_ eq. ha^−1^. System of ZTDSR-ZTW-ZTG where DSR followed by ZTW followed by ZTG showed significantly the lowest GWP. These treatments led to 8–10 % lower GWP than conventional establishment methods. Zero tillage combined with crop residue retention reduce GHG emission (Wassmann et al., 2009), increase carbon sequestration in uppermost soil layer (Li et al., 2020; Modak et al., 2019), reduce emission of methane (Metay et al., 2007) and CO_2_ (Reeves et al., 2001); and save irrigation water and diesel. Similar results were obtained by Gupta et al. (2016) where DSR-ZTW in IGPs showed a 44–47 % reduction in GWP in rice-wheat system. GWP followed ZTDSR-ZTW-ZTG < SRI-SWI-ZTG < CTMTR-ZTW-ZTG < CTDSR-ZTW-ZTG < LPTR- CTW-ZTG. Among all the treatment rice contribution in total GWP was 45–48 % in which conventional system contributed 48 % while conservation system contribution was 45 %. The current study results favoured the adoption of zero-till production system due to its benefit in terms of mitigating greenhouse gas emission from agriculture.

**Table 8:** Estimated global warming potential in kg CO_2_ equivalent ha^−1^ (due to diesel, electricity, fertilizers, herbicides, methane and nitrous oxide) under different cropping system of rice, wheat, greengram and system level (mean of 4 years).

Treatments	Details	Diesel	Electricity	Fertilizers	Herbicides	Methane	Nitrous oxide	Total GWP
RTPR-BCW-ZTG	Rice	134.00	103.54	670.50	18.76	17.42	7.39	951.59
	Wheat	117.92	57.65	670.50	20.09	0.00	4.78	870.94
	Greengram	32.16	34.51	122.68	48.40	0.00	4.06	241.81
	System							2064.34
LPTR-CTW-ZTG	Rice	134.00	103.54	670.50	18.76	17.42	7.39	951.59
	Wheat	150.08	57.65	670.50	20.09	0.00	4.78	903.10
	Greengram	32.16	34.51	122.68	48.40	0.00	4.06	241.81
	System							2096.50
CTMTR-ZTW-ZTG	Rice	160.80	103.54	670.50	48.40	17.42	7.39	1008.04
	Wheat	85.76	57.65	670.50	45.06	0.00	5.21	864.19
	Greengram	32.16	34.51	122.68	48.40	0.00	4.06	241.81
	System							2114.03
ZTMTR-ZTW-ZTG	Rice	85.76	96.42	670.50	48.40	17.42	7.39	925.88
	Wheat	32.16	57.65	670.50	45.06	0.00	5.21	810.59
	Greengram	32.16	34.51	122.68	48.40	0.00	4.06	241.81
	System							1978.28
SRI-SWI-ZTG	Rice	160.80	94.43	670.50	24.81	11.98	9.12	971.63
	Wheat	117.92	51.69	670.50	45.06	0.00	5.36	890.53
	Greengram	32.16	34.51	122.68	48.40	0.00	4.06	241.81
	System							2103.97
CTDSR-ZTW-ZTG	Rice	150.08	103.54	670.50	24.81	7.62	13.12	969.66
	Wheat	64.32	57.65	670.50	24.97	0.00	5.36	822.81
	Greengram	32.16	34.51	122.68	48.40	0.00	4.06	241.81
	System							2034.27
ZTDSR-ZTW-ZTG	Rice	32.16	103.54	670.50	48.40	7.62	10.28	872.50
	Wheat	32.16	57.65	670.50	45.06	0.00	5.36	810.73
	Greengram	32.16	34.51	122.68	48.40	0.00	4.06	241.81
	System							1925.04

### 3.10 Follow-up research and scaling-up possibilities

Intensive tillage-based conventional rice-wheat production system of the IGP of South Asia has led to a decline in productivity, input-use efficiency, and profitability; besides being energy and C-intensive. Therefore, there is an urgent need for alternative eco-friendly production systems that may be more productive and profitable; efficient user of energy, water, and carbon-based inputs. The present study demonstrated that as compared to conventional system, intensified CA-based approach (ZTDSR-ZTW-ZTG) in RWCS was more sustainable. However, DSR is not always possible in this EIGP regions that are prone to heavy rainfall at the time of sowing. This results in establishment failure due to crust formation or water logging after seeding. Mechanical seeding is either not possible or delayed due to wet soil condition. Under such situations, ZTMTR may be a good alternative for timely crop establishment. Since, weed management is a critical issue in DSR, suitable integration of pre-and post-emergence herbicides/herbicide mixture in presence of crop residue for broad-spectrum weed management needs to be developed under varying soil types and moisture regimes. More cover crops for sustainable intensification of RWCS need to be evaluated for weed suppression and allelopathic potential. As the EIGP is dominated by small and marginal farmers with lower per capita income, there is a need to strengthen the service providers in the region to scale out these profitable, but capital-intensive production options for smallholders.

## 4 Conclusion

To address the issues of declining productivity and profitability, deteriorating soil health, water scarcity, labour shortage, and climate change etc., Conservation Agriculture (CA)-based crop management practices are being developed and popularized in the IGP of South Asia. However, most of the CA technologies are confined to ZT-wheat in rice-wheat system with partial or no crop residue retention in EIGP, lacking system’s approach and crop diversification. The present study clearly demonstrates that shifting from conventional system to an intensified CA-based approach in RWCS can reduce the cost of production with similar or higher yields, and improve water productivity and profitability. The current study also suggests that the CA-based system was more energy-efficient and eco-friendly with lower system global warming potential. The information generated from this study would enhance the knowledge and strengthen the database of policymakers and researchers for promoting safer, cleaner, sustainable as well as productive climate-resilient cropping systems in the IGP of South Asia to achieve the ‘Sustainable Development Goals’. More long-term studies under varying soil types and management would be beneficial in order to increase the impact and large-scale adoption of these technologies in the EIGP.

## Declaration of Competing Interest

The authors report no declarations of interest.
