# Supplementary material for: An impact of agronomic practices of sustainable rice-wheat crop intensification on food security, economic adaptability, and environmental mitigation across eastern Indo-Gangetic Plains
Source: Field Crops Res. 2021 Jun 15;267:108164. doi: 10.1016/j.fcr.2021.108164 (PMC8146726; doi:10.1016/j.fcr.2021.108164)
Supplement: Supplementary file 1 [file mmc1.docx]

**Supplementary:**


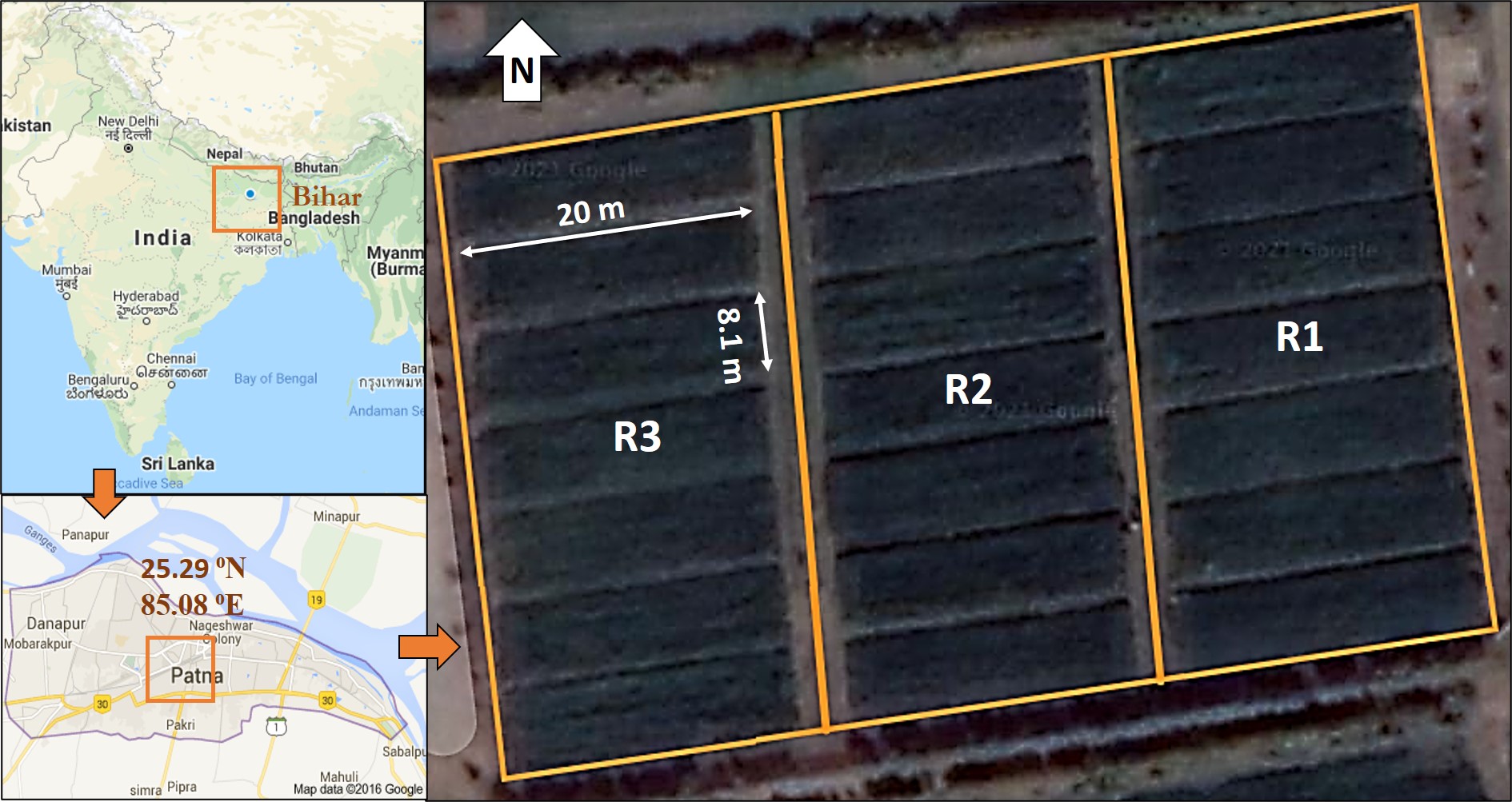


Suppl. Figure 1: Location of the experimental site.

Suppl. Table 1: Initial (year 2015) physical and chemical properties of soil at surface layer (0-15 cm) of the experimental site.

| Soil parameters | Values | Method |
| --- | --- | --- |
| pH (1:2.5 soil water) | 7.22 | pH meter with glass electrode method (Jackson, 1973) |
| E.C. (1:2.5) dSm-1 | 0.17 | Conductivity bridge method (Jackson, 1973) |
| CEC (millequivalents per 100g soil) | 14 | Ammonium acetate extraction method (Hesse, 1971) |
| Organic carbon (%) | 0.60 | 1N K2Cr2O7 solution method (Walkley and Black 1934) |
| Sand (%) | 22.00 | International pipette method (Piper 1966) |
| Silt (%) | 54.00 |  |
| Clay (%) | 24.00 |  |
| Textural class | Silty loam (Vertic Endoaqualfs) |  |
| Bulk density (Mg m-3) | 1.52 | Core sampler method (Blake and Hartge 1986) |
| Available N (kg ha-1) | 188 (low) | Alkaline potassium permanganate method (Subbiah and Asija 1956) |
| Available P (kg ha-1) | 12.9 (medium) | Ammonium molybdate extractable method (Olsen et al. 1954) |
| Available K (kg ha-1) | 137 (medium) | Ammonium acetate extraction method (Jackson 1973) |
| DTPA Iron (mg kg-1) | 14.4 | DTPA-TEA buffer method (Lindsay and Norvell 1978) using atomic absorption spectrophotometer) |
| DTPA Zinc (mg kg-1) | 0.93 |  |
| DTPA Copper (mg kg-1) | 3.63 |  |
| Infiltration rate (mm h-1) | 6.72 | Double-ring infiltrometer method (Bouwer, 1986) |
| Soil moisture at 1/3 bar (%) (FC) | 24.3 | Pressure plate apparatus (Klute, 1986) |
| Soil moisture at 15 bar (%) (PWP) | 9.8 | Pressure plate apparatus (Klute, 1986) |
| Available soil moisture (%) | 14.5 | FC (%) - PWP (%) (Klute, 1986) |
| Hydraulic conductivity (cm h-1) | 0.417 | Constant head permeameter method (Klute and Dirksen, 1986) |

Suppl. Table 2: Energy conversion factors of input and outputs used in the study (Devasenapathy et al. 2009; Parihar et al. 2017)

| Energy source | Unit | Equivalent energy (MJ unit^-1^) |
| --- | --- | --- |
| Inputs |  |  |
| Human labour |  |  |
| Adult man | Man-hour | 1.96 |
| Woman | Woman-hour | 1.57 |
| Diesel | Litre | 56.31 |
| Farm machinery | kg | 62.70 |
| Electricity | kWh | 11.93 |
| Chemical fertilizers |  |  |
| Nitrogen (N) | kg | 60.60 |
| Phosphorus (P2O5) | kg | 11.10 |
| Potash (K2O) | kg | 6.70 |
| Chemicals |  |  |
| Herbicides | kg | 254.45 |
| Insecticides | kg | 184.63 |
| Seed |  |  |
| Rice/wheat/greengram | kg | 14.7 |
| Output |  |  |
| Grain (Rice/wheat/greengram) | kg | 14.7 |
| Straw (Rice/wheat) | kg | 12.5 |

Suppl. Table 3: Greenhouse gas emission factors used for different agronomic inputs

| Input | Emission factor  (kg CO_2_ eq. per unit of input) | References |
| --- | --- | --- |
| Diesel fuel (L^-1^) | 2.68 | Kumar et al., 2018 |
| Electricity (kW h^-1^) | 0.994 | Kumar et al., 2018 |
| N (kg) | 4.95 | Kumar et al., 2018 |
| P (kg) | 0.73 | Kumar et al., 2018 |
| K (kg) | 0.545 | Kumar et al., 2018 |
| Herbicides (kg a.i.^-1^) | 24.20 | Kumar et al., 2018 |
| Methane emission factor for puddled transplanted rice (kg ha^-1^season^-1^) | 12.8 | Bhatia et al., 2013 |
| Methane emission factor for dry seeded rice (kg ha^-1^season^-1^) | 5.6 | Gupta et al., 2016 |
| Methane emission factor for system rice intensification | 8.8 | Jain et al., 2014 |

**References:**

Bhatia, A., Jain, N., & Pathak, H. (2013). Methane and nitrous oxide emissions from Indian rice paddies, agricultural soils and crop residue burning. Greenhouse Gases: Science and Technology, 3(3), 196-211.

Blake, G. R., & Hartge, K. H. (1986). Bulk density. Methods of soil analysis: Part 1 Physical and mineralogical methods, 5, 363-375.

Bouwer, H. (1986). Cylinder infiltrometer Methods of Soil Analysis Part 1. Physical and mineralogical methods.

Hesse, P. R. (1971). A textbook of soil chemical analysis (No. 631.41 H4).

Jackson, M. L. (1973). Soil chemical analysis Prentice Hall of India Ltd. New Delhi, 219-221.

Klute, A. (1986). Water retention: laboratory methods. Methods of soil analysis: Part 1 Physical and mineralogical methods, 5, 635-662.

Klute, A., & Dirksen, C. (1986). Hydraulic conductivity and diffusivity: Laboratory methods. Methods of Soil Analysis: Part 1 Physical and Mineralogical Methods, 5, 687-734.

Lindsay, W. L., & Norvell, W. A. (1978). Development of a DTPA soil test for zinc, iron, manganese, and copper. Soil science society of America journal, 42(3), 421-428.

Olsen, S. R. (1954). Estimation of available phosphorus in soils by extraction with sodium bicarbonate (No. 939). US Department of Agriculture.

Piper, C. S. (1966). Soil and plant analysis.,(Hans Publishers: Bombay, India).

Subbiah, B. V., & Asija, G. L. (1956). A rapid method for the estimation of nitrogen in soil. Current Science, 26, 259-260.

Walkley, A., & Black, I. A. (1934). An examination of the Degtjareff method for determining soil organic matter, and a proposed modification of the chromic acid titration method. Soil science, 37(1), 29-38.

Kumar, V., Jat, H. S., Sharma, P. C., Gathala, M. K., Malik, R. K., Kamboj, B. R. Yadav, A.K., Ladha, J.K., Raman, A., Sharma, D.K. & McDonald, A. (2018). Can productivity and profitability be enhanced in intensively managed cereal systems while reducing the environmental footprint of production? Assessing sustainable intensification options in the breadbasket of India. Agriculture, ecosystems & environment, 252, 132-147.

Jain, N., Dubey, R., Dubey, D. S., Singh, J., Khanna, M., Pathak, H., & Bhatia, A. (2014). Mitigation of greenhouse gas emission with system of rice intensification in the Indo-Gangetic Plains. Paddy and Water Environment, 12(3), 355-363.

Gupta, Dipak Kumar, A. Bhatia, A. Kumar, T. K. Das, N. Jain, R. Tomer, Sandeep K. Malyan, R. K. Fagodiya, R. Dubey, and H. Pathak. (2016). Mitigation of greenhouse gas emission from rice–wheat system of the Indo-Gangetic plains: Through tillage, irrigation and fertilizer management. Agriculture, Ecosystems & Environment, 230, 1-9.

Devasenapathy, P., Senthilkumar, G., Shanmugam, P.M. (2009). Energy management in crop production. Indian J Agron. 54 (1), 80-90.

Parihar CM, Jat SL, Singh AK, Majumdar K, Jat ML, Saharawat YS et al. Bio-energy, water-use efficiency and economics of maize-wheat-mungbean system under precision-Conservation Agriculture in semi-arid agro-ecosystem. Energy 2017; 119:245–56.
